# *Listeria* InlB Expedites Vacuole Escape and Intracellular Proliferation by Promoting Rab7 Recruitment via Vps34

**DOI:** 10.1128/mbio.03221-22

**Published:** 2023-01-19

**Authors:** Robert J. Cain, Mariela Scortti, Héctor J. Monzó, José A. Vázquez-Boland

**Affiliations:** a Microbial Pathogenesis Laboratory, Infection Medicine, Edinburgh Medical School (Biomedical Sciences), University of Edinburgh, Edinburgh, Scotland, United Kingdom; Washington University School of Medicine

**Keywords:** *Listeria monocytogenes*, *Listeria* virulence, internalins, InlB, intracellular parasitism, intracellular survival, intracellular proliferation, endocytic trafficking, phagosome maturation, *Listeria*-containing vacuole, vacuole escape, subversion of phosphoinositide metabolism, phosphoinositide 3-kinase, class III PI3K, Vps34, phosphatidylinositol 3-phosphate, Rab5, Rab7

## Abstract

Rapid phagosomal escape mediated by listeriolysin O (LLO) is a prerequisite for Listeria monocytogenes intracellular replication and pathogenesis. Escape takes place within minutes after internalization from vacuoles that are negative to the early endosomal Rab5 GTPase and positive to the late endosomal Rab7. Using mutant analysis, we found that the listerial invasin InlB was required for optimal intracellular proliferation of L. monocytogenes. Starting from this observation, we determined in HeLa cells that InlB promotes early phagosomal escape and efficient Rab7 acquisition by the *Listeria*-containing vacuole (LCV). Recruitment of the class III phosphoinositide 3-kinase (PI3K) Vps34 to the LCV and accumulation of its lipid product, phosphatidylinositol 3-phosphate (PI3P), two key endosomal maturation mediators, were also dependent on InlB. Small interfering RNA (siRNA) knockdown experiments showed that Vps34 was required for Rab7 recruitment and early (LLO-mediated) escape and supported InlB-dependent intracellular proliferation. Together, our data indicate that InlB accelerates LCV conversion into an escape-favorable Rab7 late phagosome via subversion of class III PI3K/Vps34 signaling. Our findings uncover a new function for the InlB invasin in *Listeria* pathogenesis as an intracellular proliferation-promoting virulence factor.

## INTRODUCTION

The Gram-positive pathogen Listeria monocytogenes is the causative agent of listeriosis, an invasive foodborne infection with severe clinical manifestations, including meningoencephalitis, septicemia, stillbirth, and neonatal sepsis (1). *Listeria* virulence depends on the ability of these bacteria to proliferate within macrophages and a variety of nonphagocytic cells. In contrast to other major intracellular pathogens, such as Mycobacterium tuberculosis or Salmonella, *Listeria* replicates in the cytosol and not in a membrane-bound vacuole (2, 3).

Within 30 min of cell entry, *Listeria* bacteria escape from acidified vesicles (4) bearing characteristics of late endosomes prior to fusion with lysosomes (Rab5 negative, Rab7 positive, and lysosomal-associated membrane protein 1 [LAMP1] negative) (5). This process is mediated by the listerial *hly* gene product, the pore-forming toxin listeriolysin O (LLO), upon its activation at acidic pH (5.5 to 6.0), aided by phospholipases PlcA and PlcB (6). Rapid multiplication ensues, fueled by hexose phosphates, which *Listeria* take up from the cytosol via the Hpt permease (7). After replication, the listerial actin-polymerizing protein ActA induces actin-based motility and cell-to-cell spread (8), allowing cytosolic *Listeria* to escape from autophagy (9) and to disseminate throughout host tissues while remaining protected from extracellular defenses (10). InlC, a member of the listerial internalin family (11), cooperates with ActA in cell-to-cell spread (12) while dampening innate immunity by inhibiting NF-κB responses (13).

Active invasion of epithelial and other normally nonphagocytic cells is critical for host barrier translocation and parenchymal tissue colonization by *Listeria*. Entry into these cells is mediated by the products of the L. monocytogenes *inlAB* operon, the surface-associated internalins InlA and InlB (14). InlA and InlB interact with specific host cell ligands, the junctional adhesion protein E-cadherin (15, 16) and the hepatocyte growth factor (HGF) receptor Met (17, 18), respectively, triggering phagocytosis. While both internalins synergize and are required for full L. monocytogenes invasiveness, InlA is thought to be specifically important for internalization into certain human epithelial cell subpopulations, whereas InlB promotes entry into a broader range of cell types (19, 20). The entry process begins with the ubiquitination of the InlA and InlB receptors, followed by recruitment of the actin cytoskeleton at clathrin-coated bacterial adhesion sites, with myosin VI providing the force for internalization (21–23). Subsequently, InlB reinforces the cortical actin polymerization cascade via activation of class I phosphoinositide 3-kinase (PI3K) downstream of Met (24), with accumulation of phosphatidylinositol 3,4,5-triphosphate (PI[3,4,5]P_3_) in the cell membrane, local recruitment of small Rho family GTPases and actin-binding proteins, and phagosome formation (reviewed in reference 25).

The *Listeria* intracellular survival-promoting determinants (LLO, PlcA, PlcB, Hpt, ActA, InlC) are coordinately regulated by the transcriptional activator PrfA (26). Consistent with their primary intracellular function, PrfA-regulated genes are weakly expressed extracellularly and selectively activated within host cells (27). Interestingly, although having an established role in cell entry, the *inlAB* locus is also controlled by PrfA (28) and its expression is activated during intracellular infection (29, 30; our unpublished results), suggesting a potential function following internalization. We investigated the possible involvement of the *inlAB* products in L. monocytogenes intracellular proliferation in human epithelial cells. Here, we report that the invasin InlB promotes early phagosomal escape, and hence bacterial intracellular proliferation, by accelerating the formation of a late-endosomal Rab7-positive compartment via subversion of class III PI3K signaling.

## RESULTS

### InlB is required for efficient intracellular proliferation.

Isogenic L. monocytogenes in-frame *inlA* and *inlB* deletion mutants were tested in human epithelial HeLa cells using gentamicin protection assays. Since the intracellular population at a given time point depends directly on the initial number of internalized bacteria, the multiplicity of infection (MOI) was adjusted such that each strain had comparable intracellular numbers at *t *=* *0 (average of ~5 × 10^4^ colony forming units [CFU]/well). In addition, the intracellular proliferation data were normalized to the bacterial counts at *t *=* *0 using an intracellular growth coefficient (IGC) (27) (see Materials and Methods).

**FIG 1:**
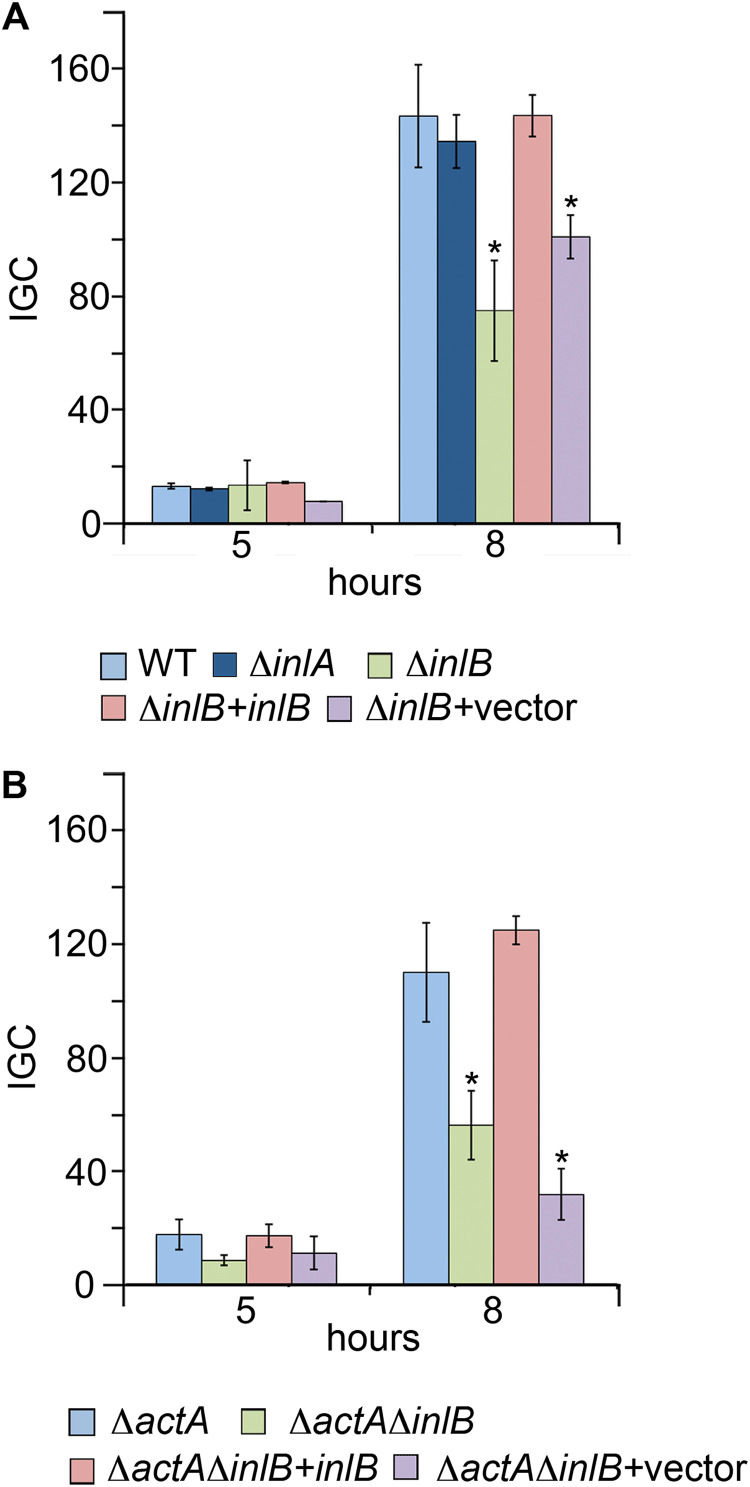
InlB is required for efficient intracellular proliferation of L. monocytogenes. Intracellular CFU of WT, Δ*inlA*, and Δ*inlB* in HeLa cells were normalized at each time point to the internalized bacterial CFU at *t *=* *0 using an intracellular growth coefficient (IGC) (see Materials and Methods). *, significant difference from WT (*P* < 0.05). (A) Data for WT, Δ*inlA*, Δ*inlB*, and *inlB*- or mock-complemented Δ*inlB* bacteria. Corresponding mean CFU/well (×10^4^) at *t* = 0 for the respective strains: 4.9 ± 1.8, 1.4 ± 0.13, 4.6 ± 1.7, 6.1 ± 4.8, and 3.9 ± 2.4. (B) Experiments in the cell-to-cell spread-null (Δ*actA*) background. Corresponding mean CFU/well (×10^4^) at *t* = 0: Δ*actA*, 4.4 ± 1.7; Δ*actA* Δ*inlB*, 3.8 ± 2.4; Δ*actA* Δ*inlB*+*inlB*, 4.3 ± 2.6; Δ*actA* Δ*inlB*+vector, 3.7 ± 2.6.

As shown in Fig. 1A, while the Δ*inlA* mutant behaved as the wild type (WT), a distinct intracellular growth defect was observed for the Δ*inlB* mutant. This was reproduced in other cell lines (human intestinal Caco-2 cells, murine J774 macrophages) (see Fig. S1 in the supplemental material) and corroborated early observations in mouse hepatocytes (31). The effect was not attributable to impaired bacterial fitness, since the Δ*inlB* mutant showed no growth defect in broth (Fig. S1B). Complementation of Δ*inlB* with the *inlB* gene on a plasmid, but not empty vector, rescued InlB production (Fig. S1C), normal cell entry (Fig. S1D), and WT intracellular proliferation (Fig. 1A), linking the observed phenotype to InlB.

10.1128/mbio.03221-22.1 FIG S1 (A) Defective intracellular proliferation of the L. monocytogenes Δ*inlB* mutant in human intestinal cells and murine macrophages. Like in HeLa cells, InlB, but not InlA, is required for efficient intracellular proliferation in (top panel) human Caco-2 enterocytes and (bottom panel) mouse J774 macrophages. IGC, intracellular growth coefficient (see the legend to Fig. 1 and Materials and Methods). *, statistically significant differences from WT (*P* < 0.01). (B to D) Characterization of the L. monocytogenes Δ*inlB* mutant. (B) The growth rate (μ parameter) and maximum growth (*A* parameter) of the L. monocytogenes Δ*inlB* mutant do not differ significantly from WT. Values were estimated from OD_600_ units in BHI at 37°C, determined every 30 min in a 96-well microplate reader (FluoStar Omega apparatus) using a logistic growth model in Prism software (GraphPad, v.9.4.1). Values are mean ± standard error of the mean (SEM) of results from four experiments. *P > *0.05 (*t* test). (C) Detection of InlB by Western immunoblotting in extracts of L. monocytogenes WT and isogenic Δi*nlB* mutant before and after complementation with *inlB* (Δ*inlB*+*inlB*) or empty vector (Δ*inlB*+vector). The blots were also processed with an anti-InlA antibody as a control. (D) Invasion efficiency of bacteria in panel A and Fig. 1A. *, statistically significant difference (*P* < 0.05). Download FIG S1, TIF file, 0.9 MB. Copyright © 2023 Cain et al. 2023 Cain et al. https://creativecommons.org/licenses/by/4.0/ This content is distributed under the terms of the Creative Commons Attribution 4.0 International license.

L. monocytogenes proliferation in a cell monolayer is dependent on both successful escape to the cytosol and actin-based spreading to adjacent cells. The contribution of cell-to-cell spread to the Δ*inlB* proliferation defect can be tested using a strain lacking ActA (8). While, as expected, overall proliferation was generally reduced in the absence of ActA due to the spread deficiency, intracellular growth was significantly diminished in a Δ*actA* Δ*inlB* double mutant compared to the Δ*actA* single mutant. Again, complementation with *inlB*, but not empty vector, reverted the proliferation defect (Fig. 1B). Together, these data indicated that the absence of InlB impairs L. monocytogenes intracellular proliferation due to effects upstream of cell-to-cell spread.

### InlB promotes early vacuole escape.

**FIG 2:**
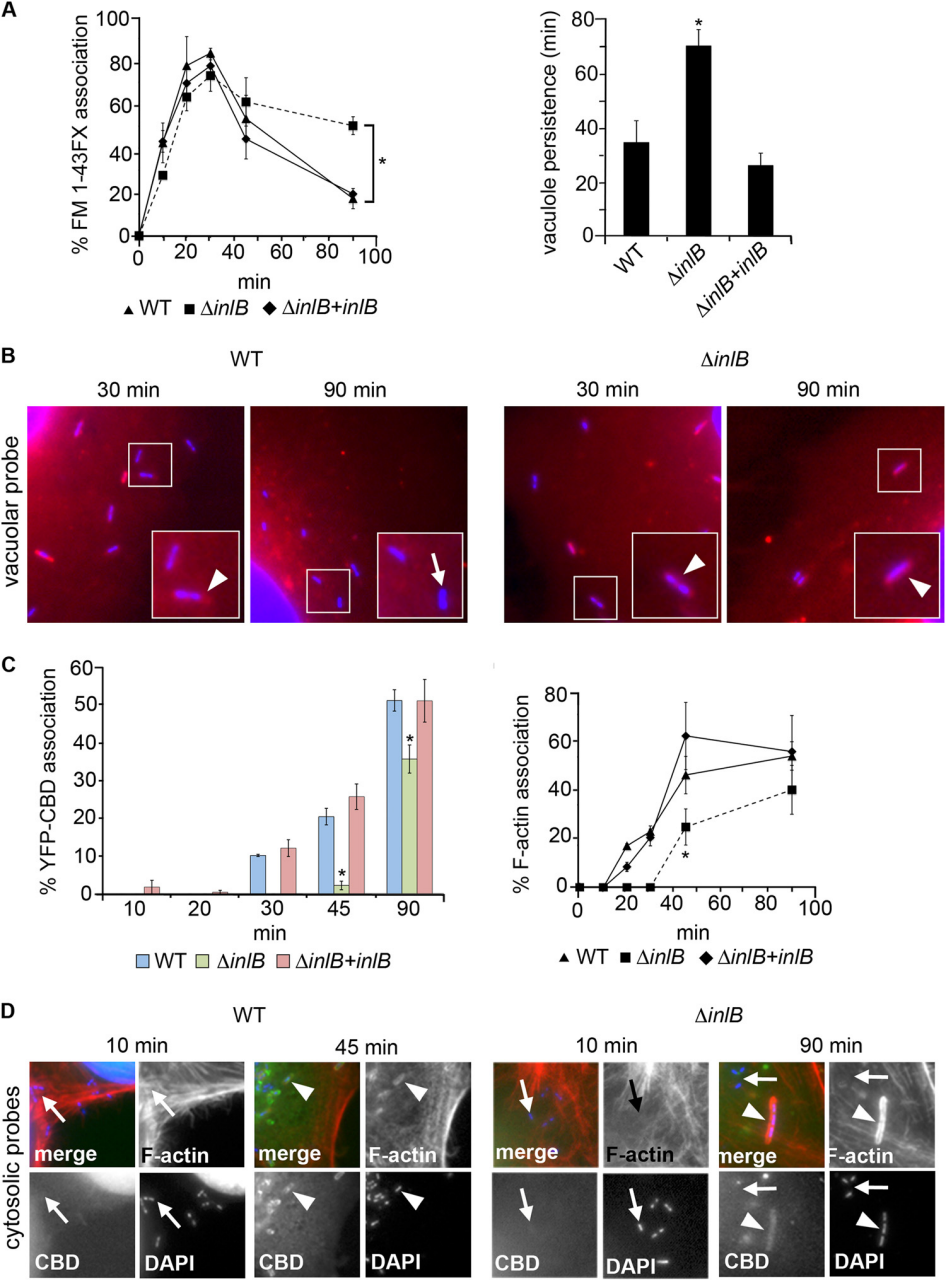
InlB promotes early vacuole escape. (A) (Left) Percentage of vacuole-associated WT, Δ*inlB*, and *inlB*-complemented Δ*inlB* bacteria determined using the constitutive membrane probe FM 1-43FX; (right) vacuolar persistence (VP) for each strain (see the text). *, significant difference from WT (*P* < 0.01). (B) Illustrative fluorescence micrographs for data in panel A. Shown are merged images of FM 1-43FX (phagosome membranes [red]) and DAPI (cell nuclei and bacteria [blue]) stains. Represented are ×2.5 enlarged areas of interest of images captured at ×630 magnification (see Fig. S2A for a complete image composite). The inset shows a further ×2.5 magnification of the area within the white rectangle (approximate total magnification, ×4,000). Arrowheads and arrows indicate examples of vacuolar and cytosolic bacteria, respectively. (C) Percentages of cytosolic WT, Δ*inlB*, and *inlB*-complemented Δ*inlB* bacteria as determined by decoration with CBD probe (left) or F-actin (right). *, significant difference from WT (*P* < 0.01 for 45 min on left panel and *P* < 0.05 for others). (D) Representative fluorescence micrographs for data in panel C. Shown are merged and individual channels for CBD (green), F-actin (Alexa Fluor 568-conjugated phalloidin [red]), and DAPI (bacteria and cell nuclei [blue]). Represented are ×2.5 enlarged areas of interest of images originally captured at ×630 magnification (complete image composite in Fig. S2B). Examples of YBD-CBD- and F-actin-associated and nonassociated bacteria are indicated by arrowheads and arrows, respectively.

The Δ*inlB* phenotype could be due either to a reduced ability to replicate within the cytoplasm or to an earlier defect in vacuole escape. To test this, the proportion of vacuole-associated and cytosolic bacteria was monitored over a 90-min infection time course in HeLa cells using two separate microscopy-based assays. Vacuole-associated bacteria were identified by colocalization with FM 1-43FX, a fluorescent membrane probe that constitutively labels endosomes and phagosomes (32, 33). The vacuole marker was associated as early as 10 min after infection with WT L. monocytogenes, with a peak at 20 to 30 min before rapidly dropping to ~20% of the bacterial population by 90 min (Fig. 2A and B). Although entering in fewer numbers (Fig. S2B), the Δ*inlB* bacteria that successfully invaded the monolayer showed similar FM 1-43FX colocalization dynamics during the first 30 to 45 min of infection. Subsequently, however, the Δ*inlB* mutant remained membrane associated in high numbers, the percentage only dropping by 20% over the rest of the time course (Fig. 2A and Fig. S2A). To quantify the phenotype while controlling for variations in cell entry rate, vacuolar persistence (VP) was calculated based on the length of time between 50% initial bacterial association and 50% dissociation with the membrane probe relative to peak values. The VP values were 34.2 ± 8.0 and 69.6 ± 5.0 min for the WT and Δ*inlB* mutant, respectively (Fig. 2A, right), suggesting the latter had a vacuole escape defect. In parallel, cytosolic bacteria were identified both through ActA-mediated F-actin accumulation (8) and decoration with a transfected fluorescent probe that binds to *Listeria* after escape to the cytosol (CBD, a listeriophage endolysin cell wall-binding domain fused to yellow fluorescent protein [YFP]) (5). Consistent with the vacuole association data, significant decoration with the CBD probe was observed later for the Δ*inlB* mutant than the WT. The dynamics of actin accumulation around bacteria mirrored the CBD probe data (Fig. 2C and D). With both approaches, the mutant phenotype reverted to WT in the *inlB*-complemented Δ*inlB* mutant (Fig. 2A and C and Fig. S2A and B). These data indicated that InlB is required for efficient vacuole escape in HeLa cells.

10.1128/mbio.03221-22.2 FIG S2 (A) InlB promotes early vacuole escape (complete image composite of Fig. 2B). Representative fluorescence micrographs illustrating association of L. monocytogenes WT, Δ*inlB* mutant, and *inlB*-complemented Δ*inlB* (Δ*inlB*+*inlB*) with phagosomes over a 90-min infection time course as determined using the constitutive endosomal membrane probe FM 1-43FX. DAPI was also used to visualize bacteria and cell nuclei. (Top panels) Wide-field ×630 merged image of the two dyes (size bar, 10 μm); (middle panels), DAPI stain alone; (bottom panels), ×2.5 magnification of the boxed areas from the top panel in color (red, FM 1-43FX-stained phagosomes; blue, DAPI-stained bacteria and cell nuclei). The inset in the bottom panel shows a further ×2.5 magnification of specific intracellular bacteria (approximate total magnification, ×4,000). Examples of vacuolar bacteria and cytosolic bacteria are indicated by arrowheads and arrows, respectively. (B) The L. monocytogenes Δ*inlB* mutant shows delayed escape to the cytosol (complete image composite of Fig. 2D). Representative fluorescence micrographs illustrate the association of L. monocytogenes WT, Δ*inlB* mutant, and *inlB*-complemented Δ*inlB* (Δ*inlB*+*inlB*) with the CBD cystosolic probe (green) over a 90-min infection time course. Samples were additionally stained with Alexa Fluor 568-conjugated phalloidin to visualize F-actin (red) and DAPI to visualize cell nuclei and bacteria (blue). (Top panels) Wide-field ×630 merged image of the three dyes in color; the size bar indicates 10 μm. Permeation of the fluorescent CBD probe (5) combined with a thicker section of the cell in the nucleus area compared to the periphery makes some nuclei show a bright CBD signal. Boxed areas of interest are shown in the bottom panel at ×2.5 magnification. Examples of CBD/F-actin-associated bacteria and nonassociated bacteria are indicated by arrowheads and arrows, respectively. Download FIG S2, TIF file, 2.6 MB. Copyright © 2023 Cain et al. 2023 Cain et al. https://creativecommons.org/licenses/by/4.0/ This content is distributed under the terms of the Creative Commons Attribution 4.0 International license.

### InlB promotes Rab7 recruitment to the *Listeria*-containing vacuole (LCV).

**FIG 3:**
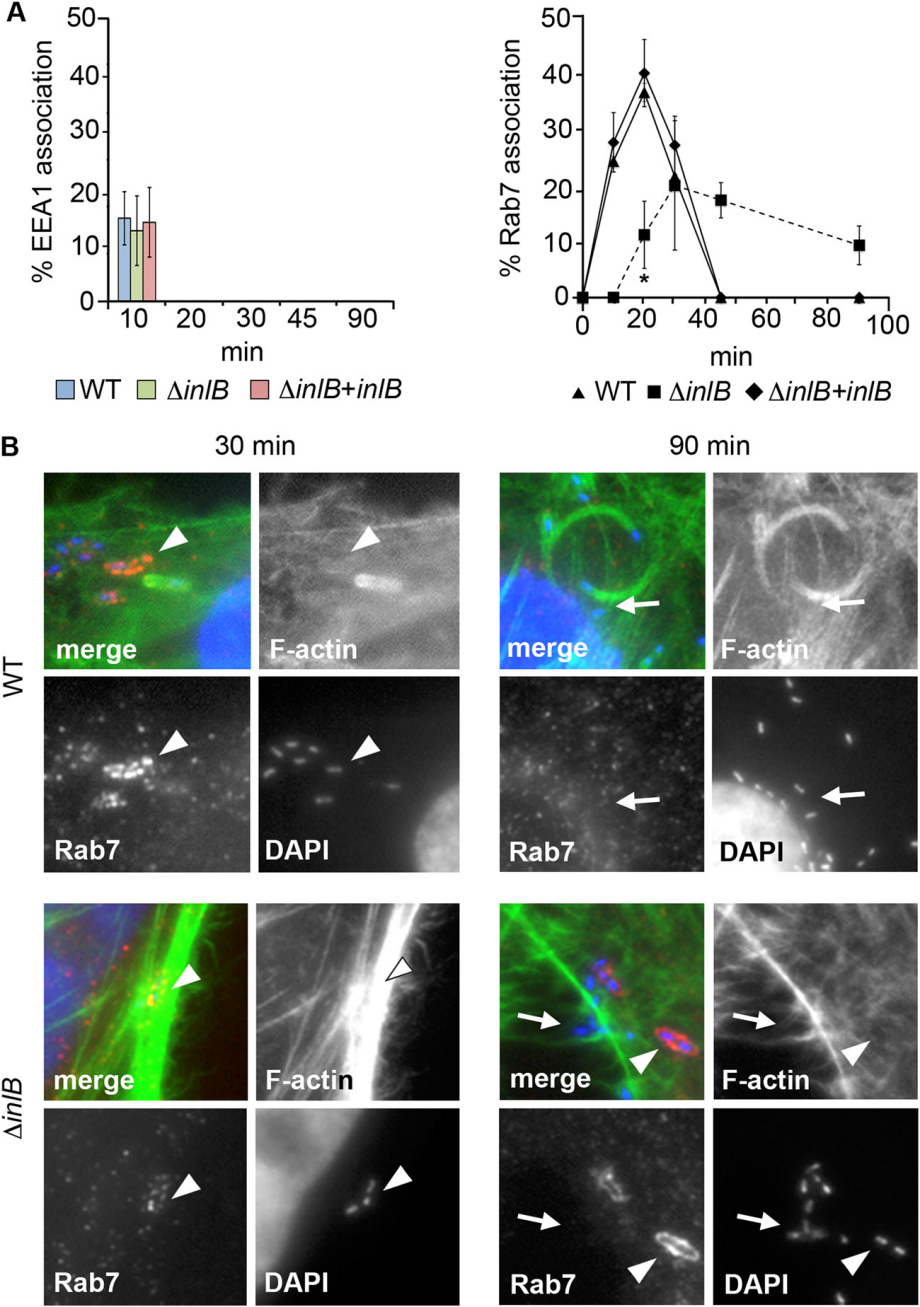
Transient recruitment of EEA1 and InlB-promoted accumulation of Rab7. (A) Percentage of EEA1- and Rab7-positive WT, Δ*inlB* and *inlB*-complemented Δ*inlB* LCVs. *, significant differences from WT (*P* < 0.05). (B) Representative fluorescence micrographs showing LCV association with Rab7 (for EEA1 see Fig. S3B). Shown are merged and individual channels for Rab7 (anti-Rab7 primary antibody and Alexa Fluor 568-conjugated anti-rabbit secondary antibodies [red]), F-actin (Alexa Fluor 488-conjugated phalloidin [green]), and bacteria/cell nuclei (DAPI [blue]). Shown are ×2.5 enlarged areas of interest of images originally captured at ×630 (complete image composite in Fig. S4B). Examples of Rab7-associated and nonassociated LCVs are indicated by arrowheads and arrows, respectively.

To explore the mechanism of Δ*inlB*’s delayed escape, different endosomal trafficking markers were probed over the infection time course in HeLa cells. Consistent with previous findings in macrophages (5), the key regulator of early to late endosome maturation, Rab5 (34, 35), was not found to localize to WT LCVs, and the same was observed for Δ*inlB* (Fig. S3A). A positive signal was clearly visible on control bacteria-containing vacuoles known to associate with Rab5 (Rhodococcus equi and Staphylococcus aureus) (Fig. S4A), excluding a technical issue with the antibody. Early endosomal antigen-1 (EEA1) (36) could be seen for both WT and Δ*inlB* bacteria, but only at 10 min after infection and for a small proportion of LCVs (~10%) (Fig. 3A, left, and Fig. S3B). Thus, EEA1 might have a role during the initial stages of *Listeria* endocytosis, as previously observed (25), but apparently only transient and independent of InlB.

10.1128/mbio.03221-22.3 FIG S3 Rab5, EEA1, and LAMP1 endosomal markers. Shown are illustrative fluorescence micrographs of HeLa cells infected with L. monocytogenes WT, Δ*inlB* mutant, and *inlB*-complemented Δ*inlB* (Δ*inlB*+*inlB*) and stained with antibody to (A) Rab5 (10 min after infection), (B) EEA1 (10 and 30 min after infection [see Fig. 3A, left panel, for quantifications]) and (C) LAMP1 (20 min after infection for WT and Δ*inlB*+*inlB* and 45 min after infection for Δ*inlB*). After the primary antibody, Alexa Fluor 568-conjugated anti-rabbit or anti-mouse secondary antibodies were used to visualize the vacuole markers (red) followed by Alexa Fluor 488-conjugated phalloidin for F-actin (green) and DAPI for cell nuclei and bacteria (blue). (Top panels) Wide-field ×630 merged images of the three dyes. Size bar, 10 μm. Boxed areas of interest are shown in the bottom panels at ×2.5 magnification. In panel B, examples of EEA1-associated bacteria and nonassociated bacteria are indicated by arrowheads and arrows, respectively. Download FIG S3, TIF file, 2.6 MB. Copyright © 2023 Cain et al. 2023 Cain et al. https://creativecommons.org/licenses/by/4.0/ This content is distributed under the terms of the Creative Commons Attribution 4.0 International license.

10.1128/mbio.03221-22.4 FIG S4 (A) Rab5 detection on Staphylococcus aureus*-* and Rhodococcus equi-containing vacuoles. S. aureus and *R. equi* are examples of bacteria whose phagosomes typically associate with Rab5 soon after phagocytosis (80, 81). S. aureus USA300 infection of HeLa cells and *R. equi* 103S infection of J774 macrophages were allowed to proceed for 10 min before fixation and processing for fluorescence microscopy. Coverslips were incubated with anti-Rab5 primary and Alexa Fluor 568-conjugated anti-rabbit secondary antibodies (red). S. aureus-infected cell monolayers were additionally stained with DAPI to visualize bacteria and cell nuclei (blue); DAPI stains *R. equi* poorly, so bacteria were visualized in these samples by phase-contrast microscopy. Examples of Rab5-positive bacteria-containing vacuoles are indicated by arrowheads. (Left) S. aureus-infected HeLa cells. (Top panels) Wide-field ×630 images. A number of bacteria (blue) can clearly be seen associating with Rab5 (red) in the merged panel (far right). (Bottom panels) Magnified ×5 areas of interest (bottom). (Right) *R. equi*-infected J774 cells. Wide-field ×1,000 images are shown on the far left. Two areas of interest, labeled a and b, are shown as ×5 enlarged panels on the right. Large vacuoles containing *R. equi* bacteria (phase dark) can clearly be seen associating with Rab5 (red). (B) Rab7 recruitment to the *Listeria* vacuole (complete image composite of Fig. 3B). Representative fluorescence micrographs illustrating association of L. monocytogenes WT, Δ*inlB* mutant, and *inlB*-complemented Δ*inlB* (Δ*inlB*+*inlB*) with Rab7 over a 90-min infection time course. Samples were stained with anti-Rab7 primary antibody and Alexa Fluor 568-conjugated anti-rabbit secondary antibodies (red), and additionally with Alexa Fluor 488-conjugated phalloidin to visualize F-actin (green) and DAPI to visualize cell nuclei and bacteria (blue). (Top panels) Wide-field ×630 merged image of the three dyes; bar indicates 10 μm. Boxed areas are shown in the bottom panel at ×2.5 magnification. Examples of Rab7-associated and nonassociated LCVs are indicated by arrowheads and arrows, respectively. Download FIG S4, TIF file, 2.7 MB. Copyright © 2023 Cain et al. 2023 Cain et al. https://creativecommons.org/licenses/by/4.0/ This content is distributed under the terms of the Creative Commons Attribution 4.0 International license.

Differences between the WT and Δ*inlB* mutant were evident, however, for the Rab7 late endosomal marker. Rab7 associated with WT LCVs soon after internalization, as previously seen in macrophages (5). The Rab7 GTPase was detected on a large proportion of phagosomes 10 min after infection, with a peak at 20 min, while it was absent 25 min later, coinciding with vacuole escape. In contrast, Δ*inlB* LCVs showed late acquisition of Rab7, with a flatter peak at 30 min followed by prolonged association up to 90 min. The phenotype could be rescued using the *inlB*-complemented mutant, indicating that it was due to InlB expression (Fig. 3A, right, and Fig. S4B). This echoed the vacuole escape experiments showing that the Δ*inlB* mutant reached the cytoplasm much later than WT (Fig. 2). Δ*inlB* was not found to colocalize with the lysosomal-associated membrane protein 1 (LAMP1), implying that although delayed in escape, the mutant was still able to avoid lysosomal fusion (Fig. S3C). Collectively, these data indicated that InlB promotes Rab7 recruitment to the LCV, and this correlated with InlB-dependent early vacuole escape.

### InlB-dependent PI3P accumulation and class III PI3K (Vps34) recruitment.

**FIG 4:**
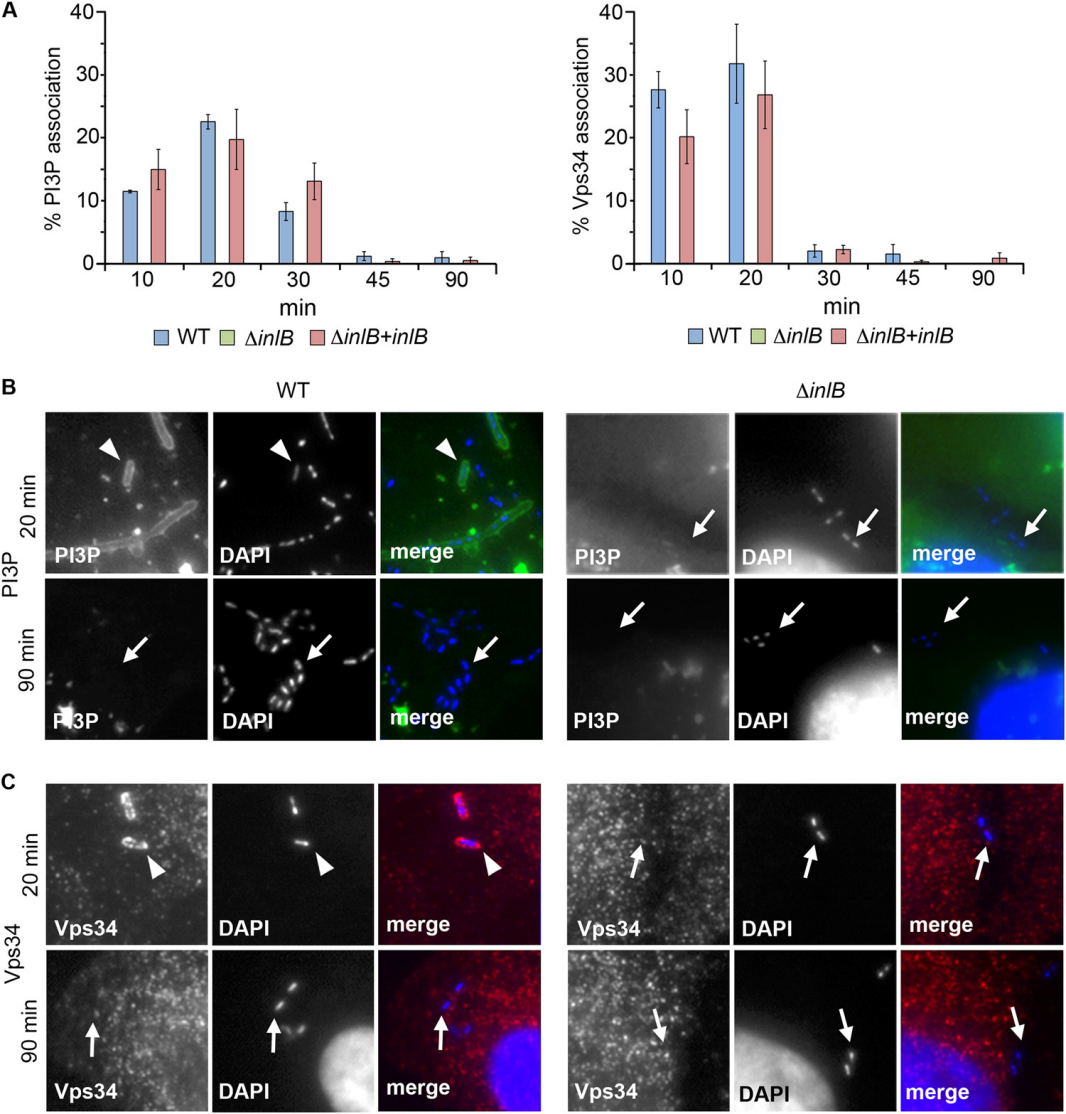
InlB-dependent Vps34 recruitment and PI3P accumulation. (A) Percentage of (left) PI3P (GFP-PX)-positive and (right) Vps34-positive WT, Δ*inlB* and *inlB*-complemented Δ*inlB* LCVs. (B and C) Representative single channel and merge fluorescence micrographs illustrating association with (B) the transfected PI3P-binding GFP-PX probe (green) or (C) Vps34 (monoclonal primary and Alexa Fluor 568-conjugated secondary antibodies [red]). Cell nuclei and bacteria were stained with DAPI (blue). Examples of PI3P- or Vps34-associated LCVs are indicated by arrowheads and nonassociated bacteria by arrows. See Fig. S5 for complete image composite.

The phosphoinositide PI3P plays a key role in the conversion of early endosomes/phagosomes into Rab7-positive vesicles (37, 38) and was previously detected in the LCV of macrophages (5). To determine whether InlB had an effect on PI3P levels in HeLa LCVs, infection time course experiments were conducted in cells expressing a fluorescent PI3P probe (GFP-PX, consisting of the PI3P-binding PX domain from the vacuolar NADPH oxidase subunit, p40*^phox^*, tagged with green fluorescent protein) (39). Accumulation of PI3P was observed after only 10 min in WT LCVs, with a peak by 20 min, declining by 30 min, and almost complete disappearance by 45 min after infection. In marked contrast, PI3P remained undetectable in Δ*inlB* LCVs during the 90-min time course. This again reverted to WT upon *inlB* complementation, indicating that, as for Rab7, PI3P accumulation was linked to InlB (Fig. 4A, left, Fig. 4B, and Fig. S5A). PI3P is primarily generated in endosomes and phagosomes by vacuolar protein sorting-34 (Vps34), the sole mammalian class III PI3K isoform (40, 41). Parallel experiments in which HeLa cells were stained with a Vps34 antibody showed Vps34 localizing to WT and *inlB*-complemented Δ*inlB* LCVs as early as 10 min after infection, while remaining absent from Δ*inlB* LCVs (Fig. 4A, right, Fig. 4C, Fig. S5B). This pattern mirrored the PI3P data, consistent with the absence of PI3P being due to a lack of Vps34 recruitment. Thus, the phagosomal escape deficiency caused by absence of InlB was associated with a lack of effective Vps34 recruitment and accumulation of its lipid product on the LCV.

10.1128/mbio.03221-22.5 FIG S5 InlB-dependent PI3P and Vps34 accumulation around the *Listeria* vacuole (complementary image composite for Fig. 4B and C). Representative fluorescence micrographs illustrate the association of L. monocytogenes WT, Δ*inlB*, and *inlB*-complemented Δ*inlB* (Δ*inlB*+*inlB*) over a 90-min infection time course with (A) PI3P (transfected PI3P-binding GFP-PX probe [green]) or (B) Vps34 (monoclonal primary- and Alexa Fluor 568-conjugated secondary antibodies [red]). All samples were additionally stained with either Alexa Fluor 568- or Alexa Fluor 488-conjugated phalloidin to visualize F-actin and DAPI to visualize cell nuclei and bacteria (blue). (Larger panels) Wide-field ×630 merged images of the three dyes. Size bar, 10 μm. Boxed areas of interest are shown on the right at ×2.5 magnification. Examples of PI3P/Vps34-associated and nonassociated bacteria are indicated by arrowheads and arrows, respectively. Download FIG S5, TIF file, 2.6 MB. Copyright © 2023 Cain et al. 2023 Cain et al. https://creativecommons.org/licenses/by/4.0/ This content is distributed under the terms of the Creative Commons Attribution 4.0 International license.

### Vps34 supports InlB-dependent intracellular proliferation but not entry.

**FIG 5:**
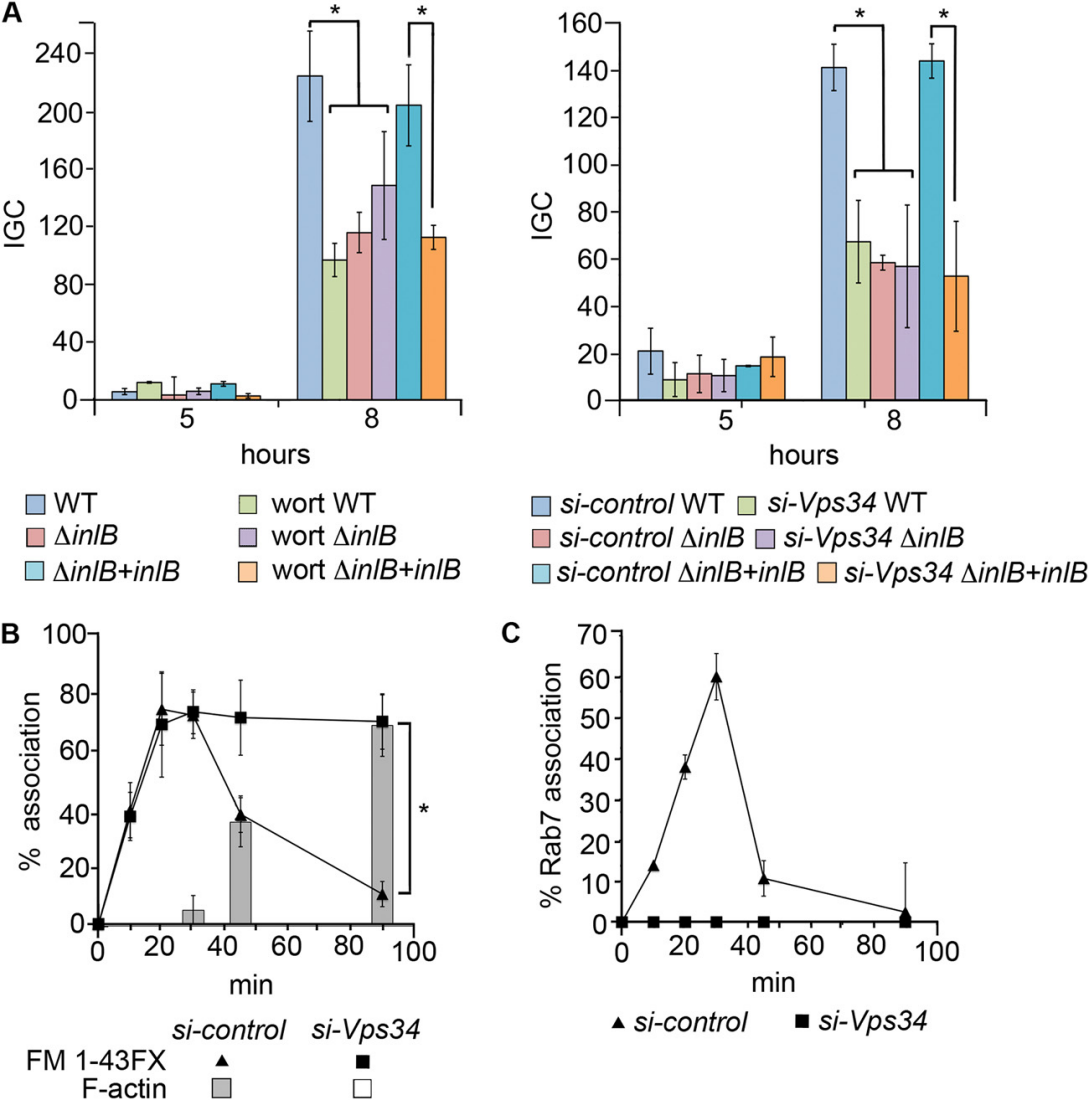
Vps34 supports InlB-dependent intracellular proliferation and vacuole escape. (A) Intracellular proliferation of WT, Δ*inlB*, and *inlB*-complemented Δ*inlB* L. monocytogenes in (left) wortmannin-treated HeLa cells (100 nM, nontreated cells with a corresponding amount of DMSO vehicle added; mean CFU/well at *t *=* *0 [×10^4^]: 3.4 ± 1.3 to 8.9 ± 3.6) or (right) cells treated with control or Vps34-specific siRNA (mean CFU/well at *t *=* *0 [×10^4^]: 1.7 ± 0.31 to 6.1 ± 0.47). IGC, intracellular growth coefficient. *, significant difference (*P* < 0.05). (B) Vacuole escape dynamics of L. monocytogenes WT in cells treated with control or Vps34-specific siRNA determined using FM 1-43FX membrane probe (vacuole-associated bacteria [line graph]) and F-actin staining (cytosolic bacteria [bars]). *, *P* < 0.01. Illustrative fluorescence micrographs are shown in Fig. S7A. (C) Same as panel B for Rab7 association (Fig. S7B).

If InlB-dependent intracellular proliferation involves class III PI3K Vps34 activity, a pan-PI3K inhibitor such as wortmannin (42) should abolish it. Wortmannin treatment of preinfected HeLa cells inhibited L. monocytogenes intracellular proliferation to an extent similar to that seen in the absence of InlB. The effect was clearly dependent on InlB, as wortmannin did not affect the intracellular proliferation of the Δ*inlB* mutant, while significant inhibition was observed with the *inlB-* but not mock-complemented mutant (Fig. 5A, left). Wortmannin had a similar InlB-dependent inhibitory effect in Caco-2 cells and J774 macrophages (Fig. S6A).

10.1128/mbio.03221-22.6 FIG S6 (A) InlB-dependent inhibion of L. monocytogenes intracellular proliferation in Caco-2 and J774 cells by wortmannin. As observed in HeLa cells, efficient intracellular proliferation in human Caco-2 enterocytes and murine J774 macrophages requires PI3K activity. IGC, intracellular growth coefficient. *, statistically significant differences from the WT (*P* < 0.05). (B to D) Wortmannin but not class I PI3K inhibitors impairs L. monocytogenes intracellular proliferation. (B) PI3K inhibitor efficacy determined by Akt phosphorylation. Lysates from cells treated with the pan-PI3K inhibitor wortmannin (100 nM) or different class I PI3K isoform-specific inhibitors (PIK-75, 100 nM; PI-103, 250 nM; TGX-221, 500 nM; AS252524, 5 μM) were immunoblotted with antibodies to phosphorylated Akt (pAktS473) and to Akt and GAPDH as loading controls. Wortmannin, PIK75 (class IA-α), and PI-103 (class IA) all showed strong inhibition of pAktS473. Total Akt levels remained unchanged, indicating this was a phosphorylation-specific effect. Such inhibition was not observed with TGX-221 (class IA-β) and AS252524 (class IB), likely due to a lower contribution to overall pAkt levels by the corresponding PI3K isoforms in HeLa cells (PI3Kα is the predominant isoform in HeLa cells, while PI3Kγ is primarily expressed in cells of the immune system) (51, 82). (C) Intracellular proliferation of L. monocytogenes in HeLa cells treated with the PI3K inhibitors described in panel B. IGC, intracellular growth coefficient (see the legend to Fig. 1 and Materials and Methods). *, statistically significant differences from the untreated control (*P* < 0.05). (D to F) siRNA knockdown of class III, but not class I or II, PI3K inhibits L. monocytogenes intracellular proliferation. (D) SDS-PAGE Western immunoblotting showing specific depletion of class I (p85), class II and class III PI3K isoforms by siRNAs using specific antibodies. Depletion of class I isoforms was also measured by Akt phosphorylation (pAktS473 antibody). GAPDH was used as a loading control. (E) Invasion efficiency of L. monocytogenes in HeLa cells depleted of different PI3K isoforms. Results are expressed as a percentage of the control. Entry into HeLa cells is mainly InlB dependent (Fig. S2B), and siRNA knockdown of the InlB signaling receptor for internalization (17) was used as a positive control. *, statistically significant differences from control siRNA-treated cells (*P* < 0.01). (F) Intracellular proliferation of L. monocytogenes in HeLa cells depleted of different PI3K isoforms, corrected for differences in entry rate by intracellular CFU normalization using an IGC (see the legend to Fig. 1 and Materials and Methods). *, statistically significant differences from control siRNA-treated cells (*P < *0.05). Download FIG S6, TIF file, 1.3 MB. Copyright © 2023 Cain et al. 2023 Cain et al. https://creativecommons.org/licenses/by/4.0/ This content is distributed under the terms of the Creative Commons Attribution 4.0 International license.

10.1128/mbio.03221-22.7 FIG S7 siRNA knockdown of Vps34 inhibits phagosomal escape and accumulation of Rab7 around the LCV. Representative immunofluorescence micrographs illustrating association of L. monocytogenes WT with (A) phagosomal membranes or (B) Rab7 over a 90-min infection time course in control or Vps34 siRNA-treated HeLa cells (quantifications shown in Fig. 5B and C, respectively). Cells were either treated with the constitutive fluorescent membrane probe FM 1-43FX to visualize phagosomes (red [A]) or incubated with anti-Rab7 primary and Alexa Fluor 568-conjugated secondary antibodies (red [B]) plus Alexa Fluor 488-conjugated phalloidin to visualize F-actin (green) and DAPI to visualize bacteria/cell nuclei (blue). The larger top panels are wide-field ×630 merged images of the three dyes. Size bar, 10 μm. Boxed areas of interest are shown below at ×2.5 magnification. Examples of phagosome membrane- (A) or Rab7- (B) associated bacteria indicated by arrowheads, of F-actin-associated (cytosolic) (A) or non-Rab7-associated (B) bacteria by arrows. Download FIG S7, TIF file, 2.8 MB. Copyright © 2023 Cain et al. 2023 Cain et al. https://creativecommons.org/licenses/by/4.0/ This content is distributed under the terms of the Creative Commons Attribution 4.0 International license.

To pinpoint the specific PI3K isoform involved, we first used isoform-specific drugs that inhibit class I PI3K. HeLa cells were treated with PIK75 (PI3Kα), TGX-221 (PI3Kβ), AS252524 (PI3Kγ) and PI-103 (PI3Kα,β,δ) (42–44), and PI3K inhibition efficacy was confirmed by Western blotting for pAktS473 (45) (Fig. S6B). None of these inhibitors significantly affected *Listeria* proliferation (Fig. S6C), indicating that the effect caused by wortmannin might be linked to non-class I PI3K. We next tested the involvement of class II and class III PI3K using small interfering RNA (siRNA) knockdown experiments. The siRNA treatments resulted in effective depletion of class II (PIKC2α and PIK3C2β; PIK3C2γ did not appear to be expressed in HeLa) and class III (Vps34) PI3K. Similar targeting of the class I PI3K regulatory subunit PIKR1 as a control resulted in a partial knockdown of p85 and a complete loss of class I PI3K activity as determined by pAktS473 Western blotting (Fig. S6D). Transfection with siRNA to the Met receptor required for InlB-mediated entry (17) was also used as an additional control. Both *Listeria* invasion and intracellular proliferation were investigated in the siRNA-transfected HeLa cells. While as expected (17, 24), both siPIKR1 and siMet significantly reduced cell invasion (60% and 80%, respectively), siRNAs to class II and class III PI3Ks had no effect, indicating they were not involved in the entry process (Fig. S6E). Conversely, in Vps34-, but not class I or class II PI3K-depleted cells, intracellular proliferation was significantly impaired for WT *Listeria* (Fig. 5A, right, and Fig. S6F), indicating that class III PI3K did indeed have a role in cell infection downstream entry. None of the siRNAs, including siVps34, had any effect on the already reduced intracellular proliferation of the Δ*inlB* mutant. As seen with wortmannin, complementation with *inlB* rescued the proliferation inhibition by siVps34 (Fig. 5A, right). Overall, these data indicated that Vps34 required InlB to exert its effect, suggesting that InlB and the class III PI3K act together to promote *Listeria* intracellular proliferation.

### Vps34 is required for Rab7 recruitment and efficient vacuole escape.

To establish whether the (InlB-dependent) effect of Vps34 on intracellular proliferation was linked to vacuole escape efficiency, siVps34- or control siRNA-treated HeLa cells were infected with WT L. monocytogenes and LCVs were monitored over 90 min using either FM 1-43FX or Rab7 antibody. Whereas bacteria appeared to rapidly disengage with LCVs in control cells (as determined by dissociation with the membrane probe and association with F-actin), they remained membrane associated for up to 90 min after infection in Vps34-depleted cells (Fig. 5B and Fig. S7A). Additionally, in contrast to control cells, no Rab7 could be seen around LCVs at any time point in siVps34-treated cells (Fig. 5C and Fig. S7B). These effects were essentially similar to, albeit more severe than, those observed with the L. monocytogenes Δ*inlB* mutant (Fig. 3A, right, and Fig. 3B), supporting the notion that InlB promotes vacuole escape by subverting LCV membrane trafficking via Vps34. These data also demonstrated the importance of Vps34 in Rab7 recruitment to the *Listeria* phagosome.

### Rapid escape requires Rab7 and is not hindered by Rab5 inhibition.

**FIG 6:**
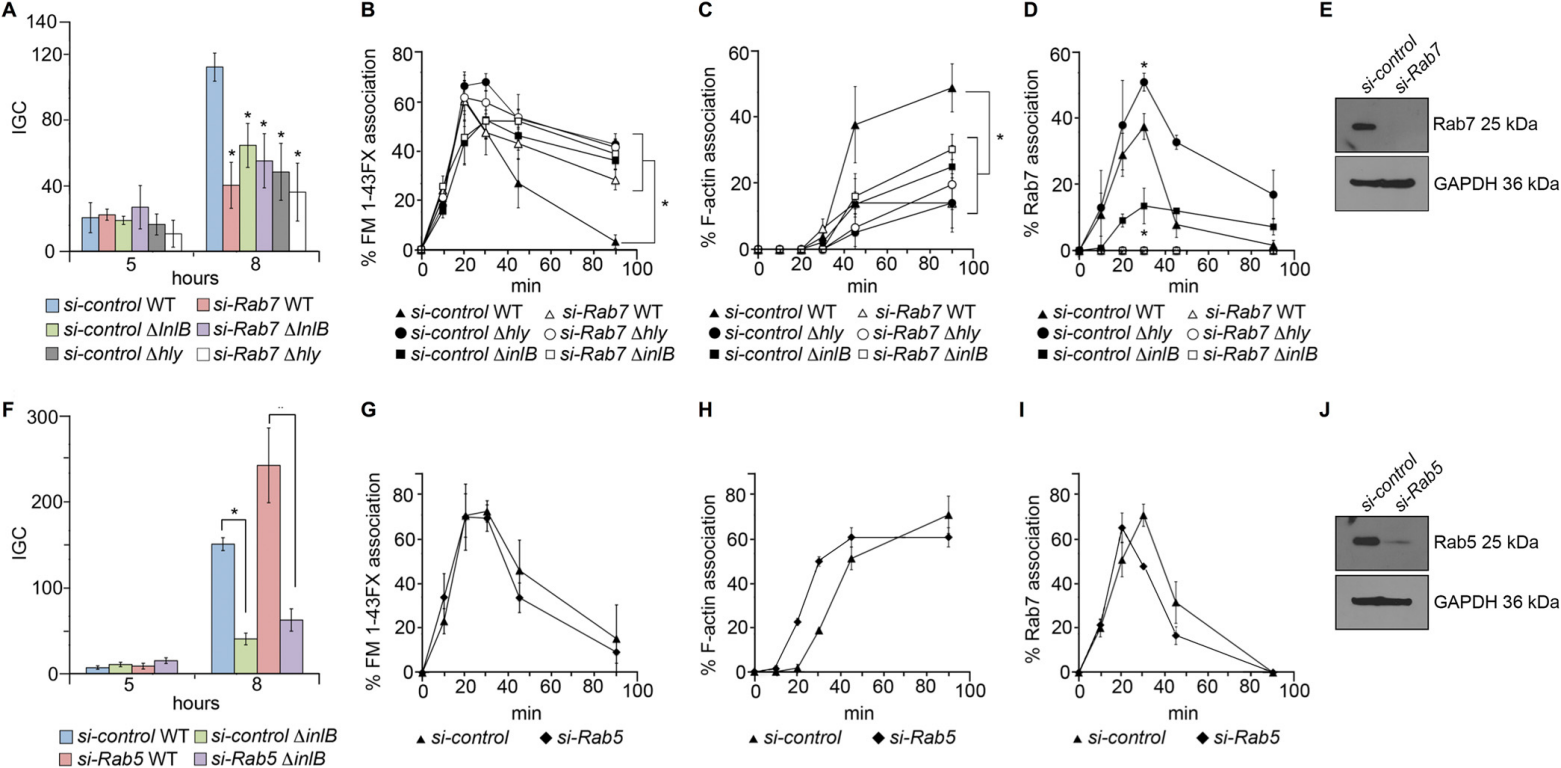
Rapid vacuole escape requires Rab7 and is not hindered by Rab5 inhibition in HeLa cells. (A) Intracellular proliferation of WT, Δ*inlB*, and Δ*hly* L. monocytogenes in Rab7-depleted or control siRNA-treated cells. IGC, intracellular growth coefficient. Mean bacterial CFU at *t *=* *0 (×10^4^): 2.7 ± 0.6 to 5.6 ± 1.2. *, significant difference from WT in control cells (*P* < 0.05). While capable of LLO-independent growth in HeLa cells, the early escape-defective Δ*hly* mutant (Fig. S8, left) shows significantly impaired intracellular proliferation, as seen with the Δ*inlB* mutant. (B, C, and D) Percentages of association for bacteria in panel A with membrane probe FM 1-43FX, F-actin, and Rab7. Illustrative fluorescence micrographs are shown in Fig. S8. *, significant differences (*P* < 0.01). (F) Intracellular proliferation of WT and Δ*inlB* L. monocytogenes in Rab5-depleted or control siRNA-treated cells. Bacterial CFU at *t *=* *0 (×10^4^): 3.8 ± 1.0 to 8.2 ± 1.2. *, significant differences (*P* < 0.01). (G, H, and I) Same as in panels B, C, and D for control and Rab5-depleted cells. Illustrative fluorescence micrographs are shown in Fig. S9C and D. (E and J) Depletion of Rab7 and Rab5 in siRNA-treated HeLa cells. Western immunoblotting with GAPDH was used as a loading control.

Listeriae appear to escape from Rab7-positive phagosomes (see reference 5 and data herein), and InlB/Vps34-promoted vacuole escape correlated with Rab7 recruitment to the LCV (this study). To test the role of Rab7 in InlB-facilitated escape, HeLa cells were transfected with control or Rab7-specific siRNAs and infected with WT or Δ*inlB* L. monocytogenes (Fig. 6A to E). Since the acidic pH of the maturing Rab7-positive phagosome is thought to be critical for efficient LCV disruption by LLO (5, 6), an isogenic Δ*hly* mutant was also included to determine whether Rab7 recruitment is linked to LLO-mediated escape. Although inefficiently, escape may occur in human epithelial cells in the absence of LLO, resulting in LLO-independent intracellular growth (46). We therefore verified that LLO was required for rapid vacuole escape under our conditions during the infection time course experiments in HeLa cells (Fig. 6A to D and Fig. S8A and B).

10.1128/mbio.03221-22.8 FIG S8 (Left panels) Rab7 is required for efficient *Listeria* phagosomal escape (representative micrographs for Fig. 6B). Association of WT and Δ*hly* L. monocytogenes with phagosomes in control or Rab7 siRNA-treated HeLa cells over a 90-min infection time course (with images for the Δ*inlB* mutant omitted as representative micrographs equivalent to control conditions are shown in Fig. S2A). Phagosomes were visualized with the constitutive membrane probe FM 1-43FX and bacteria/cell nuclei with DAPI. (Top panels) Wide-field ×630 merged color image of the two dyes (size bar, 10 μm); (middle panels) DAPI channel; (bottom panels) ×2.5 magnification of the boxed areas from the top panel (red, FM 1-43FX-stained phagosomes; blue, DAPI-stained bacteria and cell nuclei). The inset in the bottom panel shows a further ×2.5 magnification of specific intracellular bacteria (approximate total magnification, ×4,000). Examples of vacuolar membrane-associated and nonassociated bacteria indicated by arrowheads and arrows, respectively. (Right panels) Rab7 is required for efficient *Listeria* vacuole escape (representative micrographs for Fig. 6C and D). Association of WT and Δ*hly* L. monocytogenes with Rab7 in control or Rab7-treated HeLa cells over a 90-min infection time course (with images for the Δ*inlB* mutant omitted as representative micrographs equivalent to control conditions are shown in Fig. S4B). Samples were stained with anti-Rab7 primary and Alexa Fluor 568-conjugated secondary antibodies (red), Alexa Fluor 488-conjugated phalloidin to visualize F-actin (green), and DAPI to visualize bacteria/cell nuclei (blue). (Top panels) Wide-field ×630 merged images of the three dyes. Size bar, 10 μm. Boxed areas of interest are shown below at a ×2.5 magnification. Examples of Rab7-associated and -nonassociated bacteria are indicated by arrowheads and arrows, respectively. Download FIG S8, TIF file, 2.9 MB. Copyright © 2023 Cain et al. 2023 Cain et al. https://creativecommons.org/licenses/by/4.0/ This content is distributed under the terms of the Creative Commons Attribution 4.0 International license.

10.1128/mbio.03221-22.9 FIG S9 (A and B) Expression of a dominant-negative Rab5 mutant does not affect *Listeria* intracellular proliferation. (A) SDS-PAGE Western immunoblotting showing expression of the Rab5-S34N dominant-negative construct in HeLa cells. GAPDH was used as a loading control. (B) Intracellular numbers of L. monocytogenes WT and isogenic Δ*inlB* mutant in Rab5-S34N-expressing or control (empty vector) HeLa cells, normalized at each time point to the internalized bacteria at *t *=* *0 using an intracellular growth coefficient (IGC) (see the legend to Fig. 1 and Materials and Methods). Internalization was not affected by Rab5 inhibition (not shown). *, statistically significant differences (*P* < 0.05). (C and D) Rab5 depletion does not impede *Listeria* vacuole escape or Rab7 association with the LCV. (C) Representative fluorescence micrographs illustrating association of L. monocytogenes WT with phagosomes in control or Rab5 siRNA-treated HeLa cells over a 90-min infection time course (quantifications in Fig. 6G). Phagosomes were visualized with the constitutive membrane probe FM 1-43FX (red) and bacteria/cell nuclei with DAPI (blue). (Top panels) Wide-field ×630 merged color image of the two dyes (size bar, 10 μm); (bottom panels) ×2.5 magnification of the boxed areas from the top panel. The inset in the bottom panel shows a further ×2.5 magnification of specific intracellular bacteria (approximate total magnification, ×4,000). Examples of vacuolar membrane-associated and nonassociated bacteria are indicated by arrowheads and arrows, respectively. (D) Same as in panel C, but coverslips were stained with anti-Rab7 primary and Alexa Fluor 568-conjugated anti-rabbit secondary antibodies (red) and Alexa Fluor 488-conjugated phalloidin (green) to visualize association of *Listeria* with Rab7 and F-actin, respectively (quantifications in Fig. 6H and I). DAPI was additionally used to visualize bacteria/cell nuclei (blue). (Top panels) Wide-field ×630 merged image of the three dyes. Size bar, 10 μm. Boxed areas are shown in the bottom panel at ×2.5 magnification. Arrowheads and arrows indicate examples of Rab7- and F-actin-associated bacteria, respectively. Download FIG S9, TIF file, 2.6 MB. Copyright © 2023 Cain et al. 2023 Cain et al. https://creativecommons.org/licenses/by/4.0/ This content is distributed under the terms of the Creative Commons Attribution 4.0 International license.

Rab7 depletion correlated with significantly reduced intracellular growth of WT L. monocytogenes (Fig. 6A), while it did not affect internalization (not shown). Similarly reduced intracellular growth was observed for both Δ*inlB* and Δ*hly* mutants in control cells, with no significant further change in Rab7-depleted cells (Fig. 6A). Proliferation defects correlated with significantly impaired vacuole escape, as determined by fluorescence microscopy using FM 1-43FX (Fig. 6B) and F-actin staining (Fig. 6C). As expected, Rab7 was not detected in siRab7-treated cells, while in control cells, the Rab7 acquisition pattern of WT and Δ*inlB* LCVs reproduced our previous results (Fig. 6D; see also Fig. 3A, right). No defect in Rab7 acquisition, but rather a general increase, was observed for Δ*hly* LCVs, followed by a protracted association consistent with a vacuole escape deficiency (Fig. 6D). Together, these data suggest that the effects of InlB on intracellular proliferation depend on Rab7 recruitment, in turn promoting rapid, LLO-mediated vacuole escape.

Although acquisition of the early endosomal Rab GTPase Rab5 normally precedes that of Rab7 in the endosome/phagosome maturation sequence (34, 37), Rab5 could not be recognized on HeLa (our data) or mouse macrophage (5) LCVs even immediately after infection. While this may reflect only poor or transient recruitment, evidence in macrophages suggested that Rab5 might be excluded from the LCV (5). We therefore explored in a final set of experiments the role of Rab5 in *Listeria* intracellular infection. Rab5 was inhibited in HeLa cells prior to infection either by transfection with Rab5-S34N, a dominant-negative mutant in which the GTPase is locked in GDP-bound inactive form (47), or by using an siRNA pool targeting Rab5 isoforms A, B, and C (Fig. 6J and Fig. S9A). Proliferation of WT L. monocytogenes was not impaired, suggesting that entry into the cytosol was not significantly affected when Rab5 function was perturbed. The defective proliferation phenotype of the Δ*inlB* mutant also remained unaffected by Rab5 inhibition (Fig. 6F and Fig. S9B). Microscopic analysis of siRNA-treated cells confirmed that vacuole escape was not delayed in Rab5-depleted cells (Fig. 6G and H). Furthermore, no interference with Rab7 recruitment was observed in siRab5-treated cells (Fig. 6I), indicating that Rab5 does not seem to play a significant role in LCV’s maturation to the Rab7-positive stage.

## DISCUSSION

The InlB protein is a well-known *Listeria* invasin that promotes entry into nonphagocytic cells via hijacking of the ligand-dependent Met receptor endocytic pathway and downstream activation of type I PI3K (18, 24, 25, 48). Here, we provide evidence in HeLa cells that InlB has an additional key function at later stages of intracellular infection, accelerating vacuole escape, presumably through subversion of class III PI3K signaling. InlB is sufficient to mediate this process, as shown using gene complementation analysis and pharmacological and siRNA inhibition studies. The role of InlB in LCV maturation and vacuole escape is not unique to HeLa cells as similar effects were observed in Caco-2 human enterocytes (see Fig. S10). InlB has been reported to support *Listeria* infection in epithelial cells by modulating transcriptional host cell responses via SIRT2-mediated histone H3K18 deacetylation (49). Recently, InlB-induced c-Met/class I PI3K (PI3Kα) signaling has been also found to facilitate L. monocytogenes *in vivo* persistence and invasive (e.g., brain) infection by inhibiting Fas-mediated killing of infected macrophages (50). Together with these studies, our findings highlight the growing evidence that bacterial virulence factors are multifunctional, encapsulating multiple key host-cell modulatory activities to support infection.

Cell invasion permits *Listeria* to evade extracellular host defenses while vacuolar escape is essential for intracellular survival (3, 6). Since cytosolic replication relies on successful vacuole escape, optimization of the latter by InlB maximizes listerial intracellular proliferation. *In vivo* survival also crucially depends on the ability of L. monocytogenes to avoid autophagy and the cytotoxic immune response directed against infected cells via a “runaway” strategy involving ActA-mediated actin-based cell-to-cell spread after cytosolic replication (3, 10). The rapid amplification of the bacterial load is clearly an essential component of this strategy because it increases the chances of spreading before the infected cell is targeted by host defenses. Indeed, L. monocytogenes possesses a dedicated virulence factor to promote rapid intracellular replication, the hexose phosphate transporter Hpt (7). The vacuolar escape-accelerating activity of InlB here identified (together with its recently reported role in protecting infected monocytes from CD8^+^-mediated killing) (50) can therefore be seen as contributing, alongside Hpt, to provide L. monocytogenes a competitive edge against host immunity.

Strikingly, InlB targets both extracellularly and intracellularly the endocytic pathway through interference with PI3K signaling. It seems unlikely that InlB-dependent class III PI3K (Vps34)-promoted escape is directly linked to the stimulatory effect of InlB on class I PI3K during entry because Vps34 is not known to be downstream of the Met receptor. Indeed, our data show that Vps34 supports InlB-dependent intracellular proliferation but not entry. Moreover, InlB/Vps34-promoted vacuole escape was resistant to class I PI3K isoform inhibition. Rather, these two distinct InlB functions appear to be spatiotemporally coordinated, since the hijacking of the endocytic pathway occurs at two sequential levels involving stage-specific PI3K isoforms (51) coincident with consecutive steps in *Listeria* intracellular pathogenesis. After Met/class I PI3K-mediated internalization and phagocytic cup closure, a receptor-free fraction of InlB, or InlB in complex with Met before sorting for lysosomal degradation (21, 52), might direct Vps34 recruitment to the early LCV. Cotransfection experiments demonstrated colocalization of Vps34 and PI3P in InlB-positive vesicles, but we could not detect binding between InlB and Vps34 by immunoprecipitation (data not shown). This may reflect either an interaction too weak to be captured or that InlB acts upstream of Vps34.

The catalytic subunit of class III PI3K Vps34 is an effector of the small GTPase Rab5 in the endocytic pathway and localizes to nascent endosomes in complex with several regulatory subunits through interaction with membrane-bound GTP-Rab5 (41, 51). Via Vps34, Rab5 controls recruitment of PI3P-binding proteins necessary for early endosome sorting and progression to the Rab7 late endosome stage (35, 41, 51, 53, 54). Intriguingly, however, although Vsp34 was obviously present, we could not detect Rab5 in the HeLa LCVs, recapitulating previous observations in macrophages (5). The FYVE-domain (PI3P-binding) Rab5 effector EEA1 requires not only PI3P but also corecognition by GTP-Rab5 for targeting to early endosomes (35, 51, 55). EEA1 was barely detectable in the *Listeria* phagosomes regardless of significant PI3P accumulation, consistent with Rab5 being only transiently or poorly recruited to the LCV. Indeed, in contrast to Vps34 knockdown, inhibition of Rab5 did not impede Rab7 acquisition by the LCV (or *Listeria* escape/intracellular proliferation), suggesting that Rab5 has a marginal or accessory role in the process.

Our findings do not seem to support the previous proposition that L. monocytogenes actively recruits and inhibits Rab5 to block LCV maturation and delay phagolysosome fusion (56, 57). Indeed, the late endosomal marker Rab7 is rapidly recruited to the LCV after *Listeria* entry in human epithelial cells (our data) and murine macrophages (5), inconsistent with phagocytic vacuole maturation being arrested. On the contrary, L. monocytogenes appears to expedite the process via InlB-promoted recruitment of Vps34, driving LCVs to a Rab7-positive, prelysosome (LAMP1-negative) stage. This might be facilitated by diminished Rab5 levels/EEA1-mediated fusion with other (bacterium-free, Rab5-loaded) early endosomes (34, 54, 58), as this would contribute to isolating LCVs from normal endosomal traffic. The experiments using *inlB* and *hly* mutants in Rab7-depleted cells indicated that rapid (InlB-promoted) escape takes place from Rab7-positive vacuoles and that Rab7 facilitates LLO-mediated escape, likely because of the acidified intraluminal environment of the maturing endosome (6). Consequently, InlB could be seen as acting intracellularly in concert with the low-pH-activated LLO toxin to achieve efficient vacuole escape and avoid lysosomal killing.

**FIG 7:**
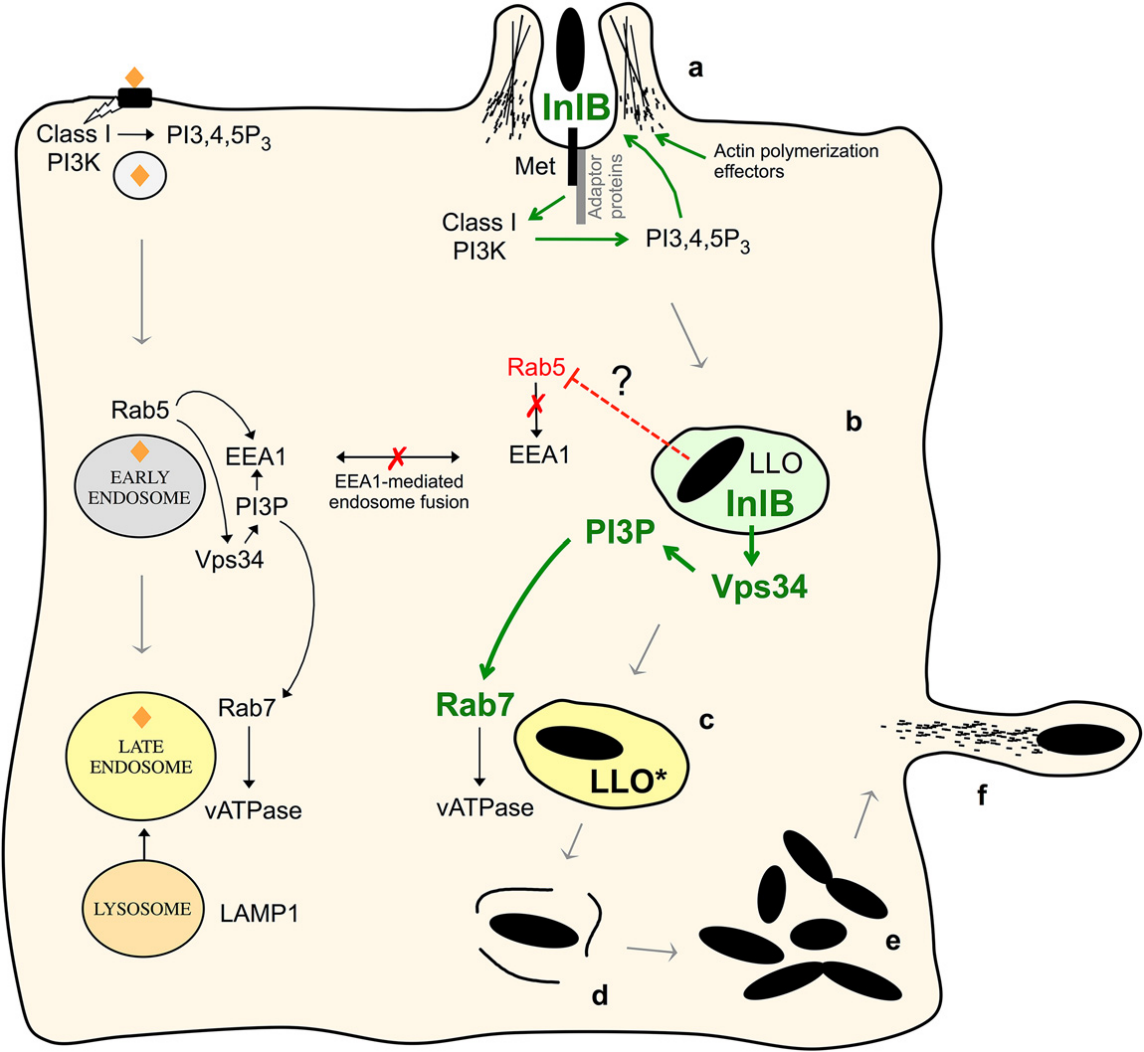
Model of sequential PI3K subversion by L. monocytogenes InlB along the endocytic pathway. At the cell surface, InlB first induces Met receptor-dependent class I PI3K activation by mimicking the natural role of HGF, leading to phagosome formation. The process involves InlB-induced autophosphorylation of the Met tyrosine kinase receptor, recruitment of adaptor proteins involved in class I PI3K recruitment/activation (Gab1, Shc, CrkII), and local accumulation of PI(3,4,5)P_3_ leading to Rac1 activation, recruitment of actin polymerization promoting proteins (Ena/VASP, WAVE, N-WASP), and activation of the Arp2/3 actin-polymerizing complex (reviewed in reference 25). After entry, InlB next modulates endosomal trafficking via activation of class III PI3K (Vps34) signaling to accelerate LLO-mediated phagosome disruption, promoting intracellular bacterial survival and proliferation. Depicted on the left is the canonical endocytic pathway leading to an acidified late endosome via recruitment of the vATPase proton pump and fusion with LAMP1-positive lysosomes; on the right is depicted the “*Listeria* pathway” with the different steps of the intracellular infection cycle. (a) InlB-dependent entry. (b) Formation of a *Listeria*-containing vacuole (LCV), typically Rab5-negative, Rab7-positive, and LAMP1-negative. Lack of detection of Rab5 in the LCV, due to either defective/transient recruitment or active exclusion (5) by unknown mechanisms, impairs EEA1 acquisition and EEA1-mediated tethering and fusion with early endosomes, contributing to isolate the LCV from normal endocytic traffic. InlB bypasses the regulatory role of Rab5 in phagosome maturation by promoting the recruitment of the Rab5 downstream effector Vps34 (class III PI3K). (c) Accumulation of the Vps34 product PI3P promotes the rapid conversion of the LCV into an acidified Rab7 late endosome-like compartment in which the pore-forming toxin LLO becomes active (LLO*). (d) Vacuole disruption occurs followed by (e) rapid bacterial proliferation in the cytosol (e) and actin-based cell-to-cell spread (f). InlB-stimulated effects are indicated in green.

The apparent lack of Rab5 recruitment to the LCV is puzzling, as the coordinated replacement of Rab5 by Rab7 appears to be key in the early-to-late endosome conversion process (34, 54, 59, 60). According to the current model, upon accumulation of PI3P, the Mon1/Sand1-Ccz1 protein complex displaces the Rab5-activating guanine nucleotide exchange factor (GEF) Rabex5, causing membrane dissociation of Rab5, and recruits/activates Rab7 through interaction with the HOPS (homotypic fusion and vacuole protein sorting) complex in association with the Rab7 GEF, Ccz1 (38, 54, 59, 61). Although GTP-Rab5 interacts with Mon1 and HOPS components (34, 38, 62), it is unclear whether Rab5 is strictly needed in the process once PI3P is generated by its downstream effector Vps34, because the Mon1-Ccz1 complex binds to PI3P (63, 64). Conceivably, therefore, L. monocytogenes might directly promote Rab7 accumulation without a sustained Rab5 input via InlB-modulated Vps34 recruitment and concomitant PI3P generation (Fig. 7). It is worth noting, however, that even in the absence of InlB, and thus of detectable Vps34 and PI3P accumulation, Rab7 acquisition still occurred in the LCV, albeit more slowly. This observation is consistent with previous data showing significant Rab7 recruitment to phagosomes in wortmannin-treated cells (37), implying that the phagosome maturation process can be, at least partially, independent of Vps34 activity (65). Nevertheless, our siRNA experiments support a crucial role for Vps34 because its depletion appeared to prevent Rab7 recruitment to the LCV. Since via a regulatory loop Vps34 negatively regulates Rab5 during endosome maturation (66), InlB-promoted Vps34 recruitment itself might have a role in the lack of detection of Rab5 in the LCV. Multiple mediators and interactors are involved in the intricate regulation of endosome trafficking (41, 51, 54, 59, 60), and further research is needed to clarify the mechanism underlying the apparent Rab5 exclusion and how InlB promotes Vps34 and Rab7 recruitment in the LCV.

Avoidance of lysosomal killing by vacuolar pathogens often involves interference with class III PI3K/PI3P signaling, but the specific underlying mechanism differs for each pathogen (67, 68). M. tuberculosis, for example, causes early phagosome maturation arrest through depletion of PI3P by the secreted SapM phosphatase as well as inhibition of Vps34 by mycobacterial cell envelope components. Rab5 is retained, but EEA1 and Rab7 are excluded, blocking acquisition of the acidifying vacuolar proton pump ATPase and lysosomal fusion (69–72). Salmonella, in contrast, promotes homotypic fusion of PI3P-enriched, Rab5- and EEA1-positive vesicles to form a large vacuole in which the bacteria proliferate. This is mediated by the type III secreted effector SopB, which promotes PI3P generation by dephosphorylating phosphatidylinositol di- and triphosphates and through recruitment of Rab5 and Vps34 activation (73, 74). Here, we show that L. monocytogenes, via InlB, promotes the recruitment of Vps34 and induces PI3P generation on the LCV, boosting the formation of an escape-favorable Rab7 late phagosome. Our data offer a novel example of bacterial modulation of endosomal trafficking, unexpectedly in a cytosolically replicating pathogen that only very transiently interacts with the phagocytic vacuole after internalization.

## MATERIALS AND METHODS

### Bacteria, plasmids, antibodies, and reagents.

Isogenic derivatives of L. monocytogenes serovar 4b clinical isolate P14 with constitutive *in vivo*-like virulence gene expression (75) were used in this study. The construction of Δ*inlA* and Δ*inlB* in-frame deletion mutants is described below, the Δ*actA* and Δ*hly* mutants have been reported elsewhere (27, 76). The *R. equi* 103S and S. aureus USA300 strains used as controls for Rab5 detection in bacterial phagosomes were from our bacterial isolate collection and R. Fitzgerald’s laboratory (Roslin Institute, University of Edinburgh), respectively. Mammalian expression plasmids pEYFP-CBD, encoding the *Listeria* cell wall-binding cytosolic probe, and pGFP-iPX, encoding the PI3P-binding probe, were kindly provided by J. Swanson’s lab (University of Michigan Medical School, Ann Arbor, MI, USA) and the L. Stephen/P. Hawkins’s lab (Babraham Institute, Cambridge, United Kingdom), respectively (5, 39). The plasmid pHinlB used for complementation of the Δ*inlB* mutant is a derivative of the E. coli Gram-positive shuttle vector pHPS9 (77) containing the *inlB* gene. The insert was prepared by PCR amplification of *inlB* and upstream *inlAB* operon regulatory regions from *L. monocytogenes* PAM14 Δ*inlA* genomic DNA using oligonucleotides mainl13SalI13 (ACGCGTCGACGAACATAAAGGGTAGAGG) and inlBBamHI (CGGGATCCCGATTCTTGCTAGACCACC), which carry SalI and BamHI restriction sites (underlined), respectively. After cloning into the pTOPO cloning T-vector (Invitrogen), the SalI/BamHI insert was transferred to pHPS9.

### Reagents, antibodies, and oligonucleotides.

Chemicals were from Sigma-Aldrich unless stated otherwise. Class I PI3K inhibitors PIK-75, TGX-221, AS-604850, and PI-103 were from Cayman Chemicals. FM 1-43FX membrane probe was from Life Technologies. Antibodies to Akt, pAktS473, Rab7, Rab5, EEA1, and Vps34 were from Cell Signaling Technology, GAPDH (glyceraldehyde-3-phosphate dehydrogenase) from Chemicon, p85 from Millipore, PI3KC2α and PI3KC2β from BD Biosciences, PI3KC2γ from Abgent, LAMP1 (H4A3) from Santa Cruz, and horseradish peroxidase (HRP)-conjugated-mouse and rabbit antibodies from Amersham. Specific antibodies to InlA and InlB were kindly provided by P. Cossart, Institut Pasteur, Paris, France. Oligonucleotides for siRNA knockdowns were from Dharmacon. Pools of four individual siRNA oligonucleotides were used to target each of the human PI3K genes PIK3R1 (D-003020-10, D-003020-11, D-003020-26, D-003020-27) PIK2CA (D-006677-01, D-006771-02, D-006771-03, D-006771-04), PIK2CB (D-006772-01, D-006772-02, D-006772-03, D-006772-04), PIK2CG (D-006773-01, D-006773-02, D-006773-03, D-006773-04) and PIK3C3 (D-005250-01, D-005250-02, D-005250-03, D-005250-04). Pools of four oligonucleotides were also used for Rab5A (D-004009-01, D-004009-02, D-004009-03 D-004009-04), Rab5B (D-004010-1, D-004010-2, D-004010-3, D-004010-4), Rab5C (D-004011-1, D-004011-2, D-004011-3, D-004011-4), and Rab7 (D-010388-01, D-010388-02, D-010388-03, D-010388-04), while the HGF receptor Met was targeted with oligonucleotide D-003156-13. Oligonucleotide primers for PCR or genetic constructs were purchased from Sigma or Metabion (Martinsried, Germany).

### General DNA techniques.

Restriction enzymes were used according to the manufacturer’s instructions (Promega and NEB). Chromosomal DNA from *Listeria* was extracted and purified as previously described (75). Plasmid DNA was extracted from E. coli using the Qiagen plasmid purification kit and was introduced into E. coli or L. monocytogenes by electroporation using a GenePulserXcell apparatus (Bio-Rad). PCR was carried out with *Taq* DNA polymerase (Biotools, Madrid, Spain) for detection/mapping purposes or high-fidelity ProofStart DNA polymerase (Qiagen) for plasmid or mutant constructions. The reaction mixtures contained 100 ng of DNA template, 200 μM deoxynucleoside triphosphates (dNTPs), 0.25 μg oligonucleotide primers, 2.5 mM MgCl_2_, a suitable amount of polymerase buffer, and 1 U of polymerase per kb in a 25-μL volume. The standard amplification program was 3 min at 94°C, followed by 30 cycles of 15 s at 95°C, 30 to 60 s at 48 to 58°C, and 1 to 3 min at 72°C, and a final 3 min at 72°C. PCR products were purified with the Qiagen PCR purification kit. DNA sequences were determined on both strands.

### Construction of L. monocytogenes *inlA* and *inlB* in-frame deletion mutants.

Chromosomal gene deletions were generated by allelic exchange via homologous recombination as previously described (76). For the Δ*inlA* mutant, the recombinogenic plasmid was constructed by in-frame ligation of two PCR fragments containing small portions of the 5′- and 3′-terminal regions of the *inlA* gene plus part of the upstream and downstream flanking regions, respectively. Oligonucleotides inlA1 (ATTTGGATCCTAAAGGGTAGAGG) and inlA2 (CATACCCCGGGGCCAAATACT) carrying BamHI and XmaI/SmaI sites, respectively (underlined), were used to PCR amplify a 643-bp fragment from the 5′ region of *inlA*, including the first 72 bp of the gene. Similarly, oligonucleotides inlA3 (TCACCCGGGAATTCAGCTAGC) and inlA4 (TGAACGGATCCAATATCACTATTAT) carrying XmaI/SmaI and BamHI sites, respectively, were used to amplify a 709-bp fragment from the 3′ region of *inlA*, including the last 174 nucleotides of the gene. The two PCR products were digested with XmaI and ligated, and the ligation product was used as a template for PCR amplification with oligonucleotides inlA1 and inlA4. The resulting amplicon was inserted into the pTOPO T-vector (Invitrogen) and then the BamHI fragment containing the Δ*inlA* construct was transferred to the thermosensitive shuttle vector pMAD (78) to give the recombinogenic plasmid pMΔ*inlA*. The in-frame-deleted allele for the Δ*inlB* mutant was generated by “recombinant PCR” (79). Oligonucleotide primers inlB1 (TCCTGTGGATCCACCAACAACT) and inlB2R (TACCGGAACTTTTGTCACTAGATCCGTCACAC) amplified a 573-bp fragment from the 5′ region of *inlB*, including the first 213 bp of the gene, and primers inlB3R (GATCTAGTGACAAAGTTCCGGTAGTAGATAGC) and inlB4 (GTGATGGATCCCACATTTTGGC) were used to amplify the 3′ region of *inlB*. Primers inlB1 and inlB4 carried BamHI sites, and primers inlB2R and inlB3R had complementary sequences (underlined). The two PCR products were fused by splicing overlap extension using oligonucleotides inlB1 and inlB4, and the recombinant PCR product was processed as described above to produce the pMAD-based recombinogenic plasmid pMΔ*inlB*.

### Mammalian cell cultures and transfections.

HeLa cells (and Caco-2 and J774 cells for some control experiments) were sourced from ATCC, passaged at no more than 70% confluence, and used between passages 3 and 15. Cells were cultured in 24-well plates or on 13-mm glass coverslips at 37°C under 5% CO_2_ in DMEM (Dulbecco's modified Eagle's medium supplemented with 10% fetal calf serum [FCS] and l-glutamine [Invitrogen]) without antibiotics, as previously described (45). HeLa cells were seeded at a density of 3.5 × 10^4^ or 5 × 10^4^ cells per well (24-well plates) or coverslip 24 h prior to transfection with siRNA or plasmid, respectively. Plasmid DNA or siRNAs diluted in optiMEM without FCS were premixed with optiMEM-diluted Lipofectamine 2000 reagent (Invitrogen) as per the manufacturer’s instructions. Cells were transfected for 12 h in 1 mL DMEM, giving a final concentration of 80 to 200 nM oligonucleotide or 1 to 5 μg/mL plasmid DNA. Transfected cells were incubated for 24 h prior to infection, fixation for immunofluorescence microscopy, or production of cell lysates. Plasmid transfection efficiency was typically >85%.

### Cell infections.

Culture plates containing ≈1 × 10^5^ cells per well were inoculated with bacterial suspensions in DMEM and immediately centrifuged (180 × *g*, 3 min) to synchronize infection. Internalization and intracellular proliferation assays were carried out using a gentamicin protection assay as previously described (27). After centrifugation, inoculated plates were incubated for 30 min (15 min for J774 macrophages) and washed several times with Dulbecco’s phosphate-buffered saline (DPBS) (Invitrogen) followed by a further incubation in DMEM containing 100 μg/mL gentamicin for 30 min to kill the remaining extracellular bacteria. Samples were taken at this point to determine invasion (*t *=* *0), and medium was replaced with fresh medium containing 10 μg/mL gentamicin to prevent extracellular bacterial growth. Where applicable, PI3K inhibitors were added at this stage diluted in 0.1% dimethyl sulfoxide (DMSO). At the sampling time points, cells were washed in DPBS, lysed in 10 mM Tris-HCl (pH 7.4), 1% Triton X-100, and viable bacteria plate counted. Intracellular proliferation data were normalized using an intracellular growth coefficient to correct for variation in cell entry using the formula IGC = (CFU*_t_*_n_ − CFU*_t_*_0_)/CFU*_t_*_0_ (27).

### Western blotting.

HeLa cells were processed for immunoblotting as detailed by Cain et al. (45), and bacterial proteins were prepared as previously described (76). Immunoreactive proteins were visualized using the ECL enhanced chemiluminescence detection system (Amersham).

### Fluorescence microscopy.

Coverslips for time course quantifications were collected 10, 20, 30, 45, and 90 min after plate centrifugation, thoroughly washed four times in warm PBS to remove extracellular bacteria, fixed with 3.7% (wt/vol) paraformaldehyde, and processed as described by Cain et al. (45). For time points 45 and 90 min, 100 μg/mL gentamicin was added to the medium 30 min after infection to prevent extracellular growth. Alexa Fluor 488- or 568-conjugated anti-rabbit or anti-mouse IgG secondary antibodies (1 h) were used for immunofluorescence staining, followed by Alexa Fluor 568- or 488-conjugated phalloidin (Invitrogen) and DAPI (4′,6-diamidino-2-phenylindole) (Sigma) (20 min) to visualize F-actin and bacterial DNA/cell nuclei, respectively. To determine vacuole-associated bacteria, cells were incubated with 2 μM of the fixable fluorescent membrane probe FM 1-43FX (Life Technologies) in DMEM for 45 min, washed twice, and incubated with fresh DMEM for 30 min before infection. Bacteria that escaped from the vacuole were identified based on their association with either F-actin (46) or a transfected listerial cell wall-binding fluorescent cytosolic probe (yellow fluorescent protein [YFP]-CBD) (5). Images were acquired at room temperature using a Leica CTR-6000 fluorescence microscope with a 63× oil immersion objective, collecting data as a z-stack, which were later flattened by deconvolution. Images were processed using Leica LAF software (Leica) and ImageJ (NIH; http://rsbweb.nih.gov/ij/), and figures were assembled using Adobe Photoshop.

### Statistics.

In all cases, data are shown as the mean ± standard error of the mean (SEM) of values from a minimum of three independent experiments performed in triplicate. Data for image-based quantifications were collected from 60 to 70 bacteria/vacuoles across five randomly selected microscopic fields per time point per condition in each of three independent experiments. The statistical significance of data was determined by Student *t* tests using Prism software (http://www.graphpad.com/scientific-software/prism/).

10.1128/mbio.03221-22.10 FIG S10 InlB promotes Vps34 recruitment and early vacuole escape in human Caco-2 enterocytes (see also Fig. S1A). (A) Association of L. monocytogenes WT and Δ*inlB* mutant with Vps34, Rab7, and F-actin over a 90-min infection time course. *, statistically significant differences from WT (*P* < 0.05). (B) Representative fluorescence micrographs for Vps34 association. Staining was with monoclonal anti-Vps34- and Alexa Fluor 568-conjugated secondary antibodies (red), Alexa Fluor 488-conjugated phalloidin (F-actin [green]), and DAPI (bacteria/cell nuclei [blue]). (Top panels) Wide-field ×630 merged images of the three stains. Size bar, 10 μm. Boxed areas are shown below at ×2.5 magnification (merge and individual channels). Arrowheads and arrows indicate examples of Vps34-associated and -nonassociated bacteria, respectively. (C) Representative fluorescence micrographs for Rab7 and F-actin association. Coverslips were processed as in panel B, except that anti-Rab7 primary antibody was used. Arrowheads and arrows indicate examples of Rab7-associated and nonassociated bacteria, respectively. Note among the latter that at 90 min after infection, most WT L. monocytogenes cells are surrounded by F-actin rings, indicating cytosolic location after vacuole escape; in contrast, only a small proportion of Δ*inlB* bacteria are F-actin associated, consistent with a delayed escape. Download FIG S10, TIF file, 2.8 MB. Copyright © 2023 Cain et al. 2023 Cain et al. https://creativecommons.org/licenses/by/4.0/ This content is distributed under the terms of the Creative Commons Attribution 4.0 International license.
