# Supplementary figures and images for: Listeria InlB Expedites Vacuole Escape and Intracellular Proliferation by Promoting Rab7 Recruitment via Vps34
Source: mBio. 2023 Jan 19;14(1):e03221-22. doi: 10.1128/mbio.03221-22 (PMC9973280; doi:10.1128/mbio.03221-22)

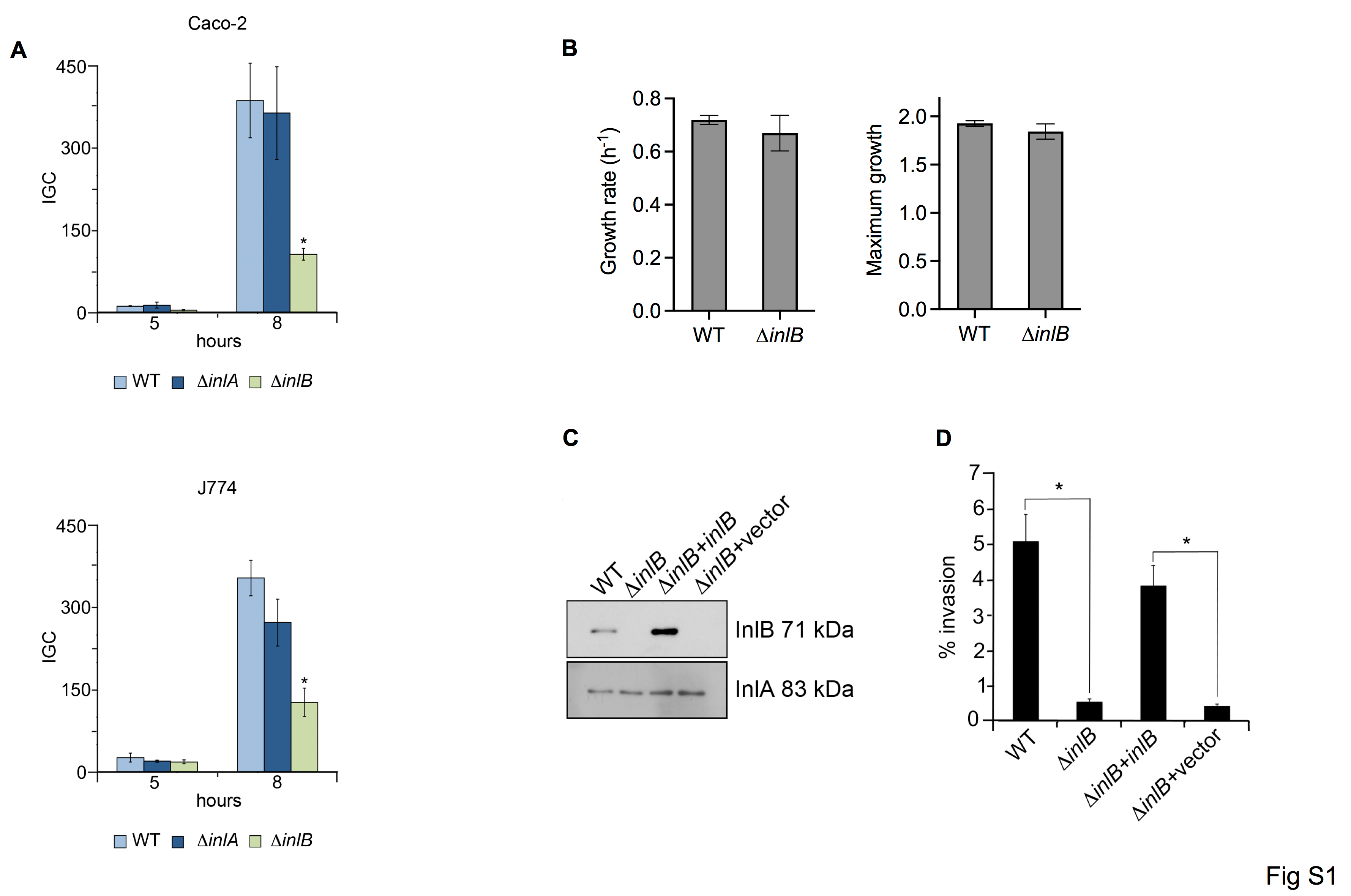

Supplement: FIG S1 [file mbio.03221-22-s0001.tif]

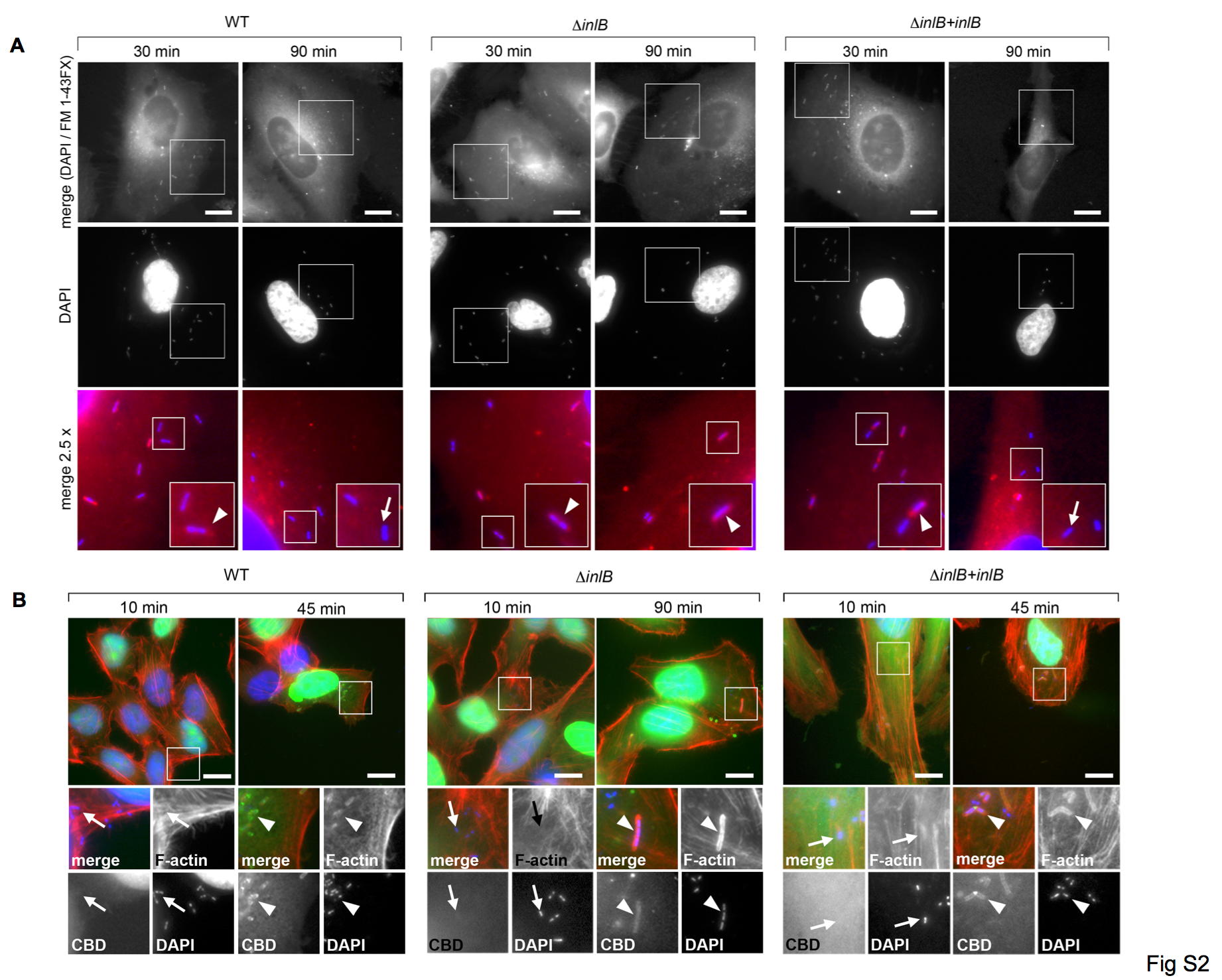

Supplement: FIG S2 [file mbio.03221-22-s0002.tif]

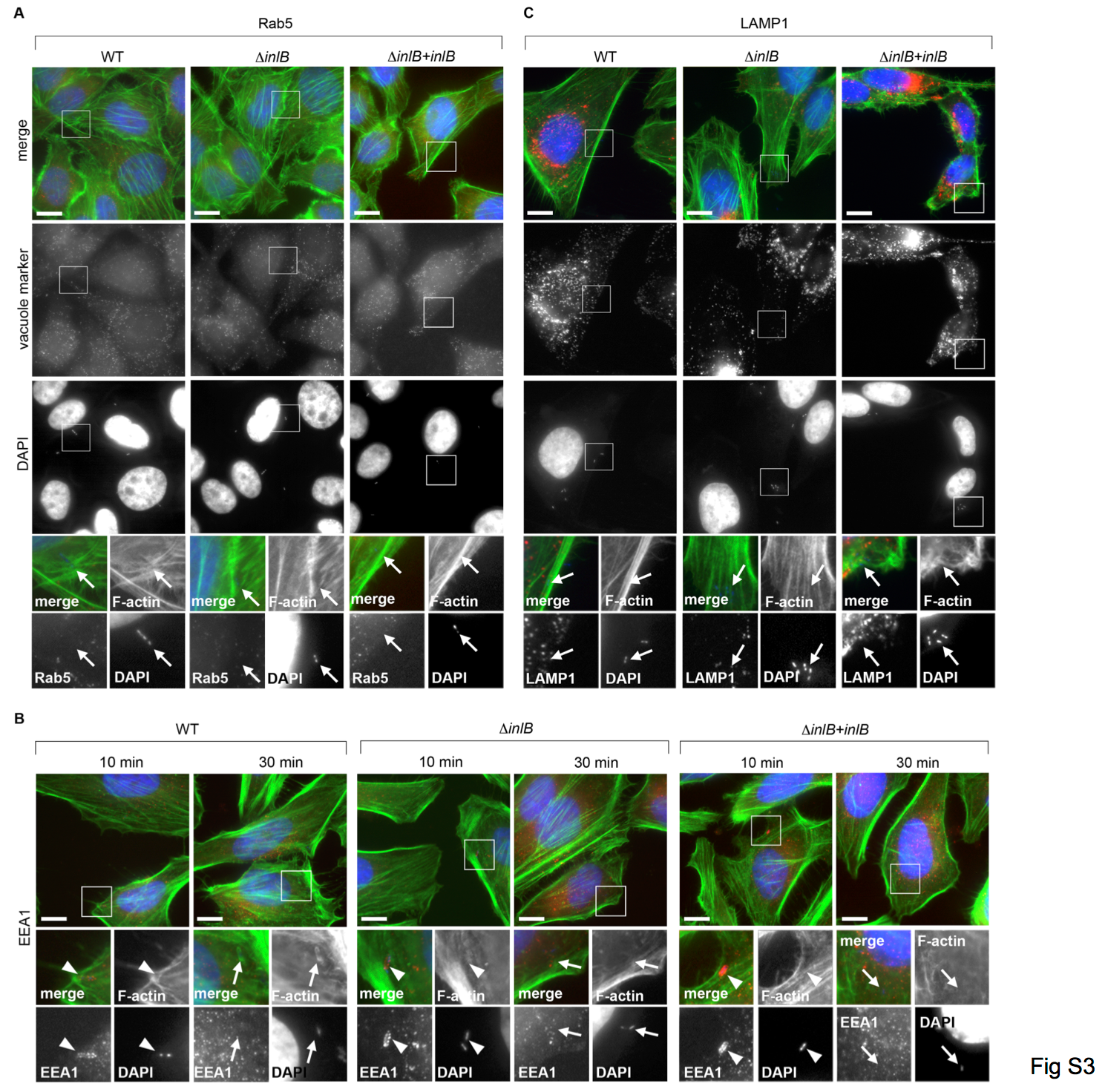

Supplement: FIG S3 [file mbio.03221-22-s0003.tif]

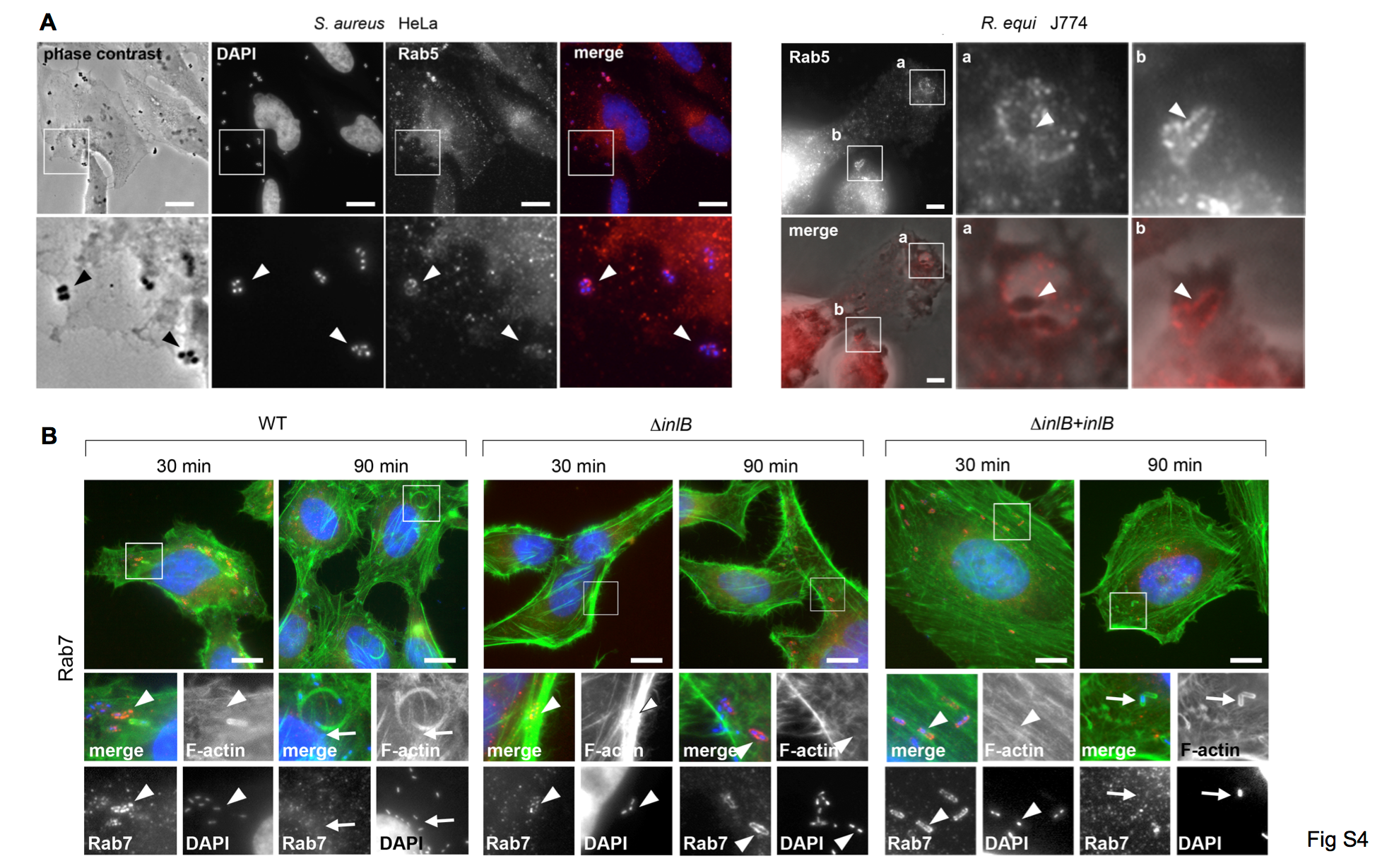

Supplement: FIG S4 [file mbio.03221-22-s0004.tif]

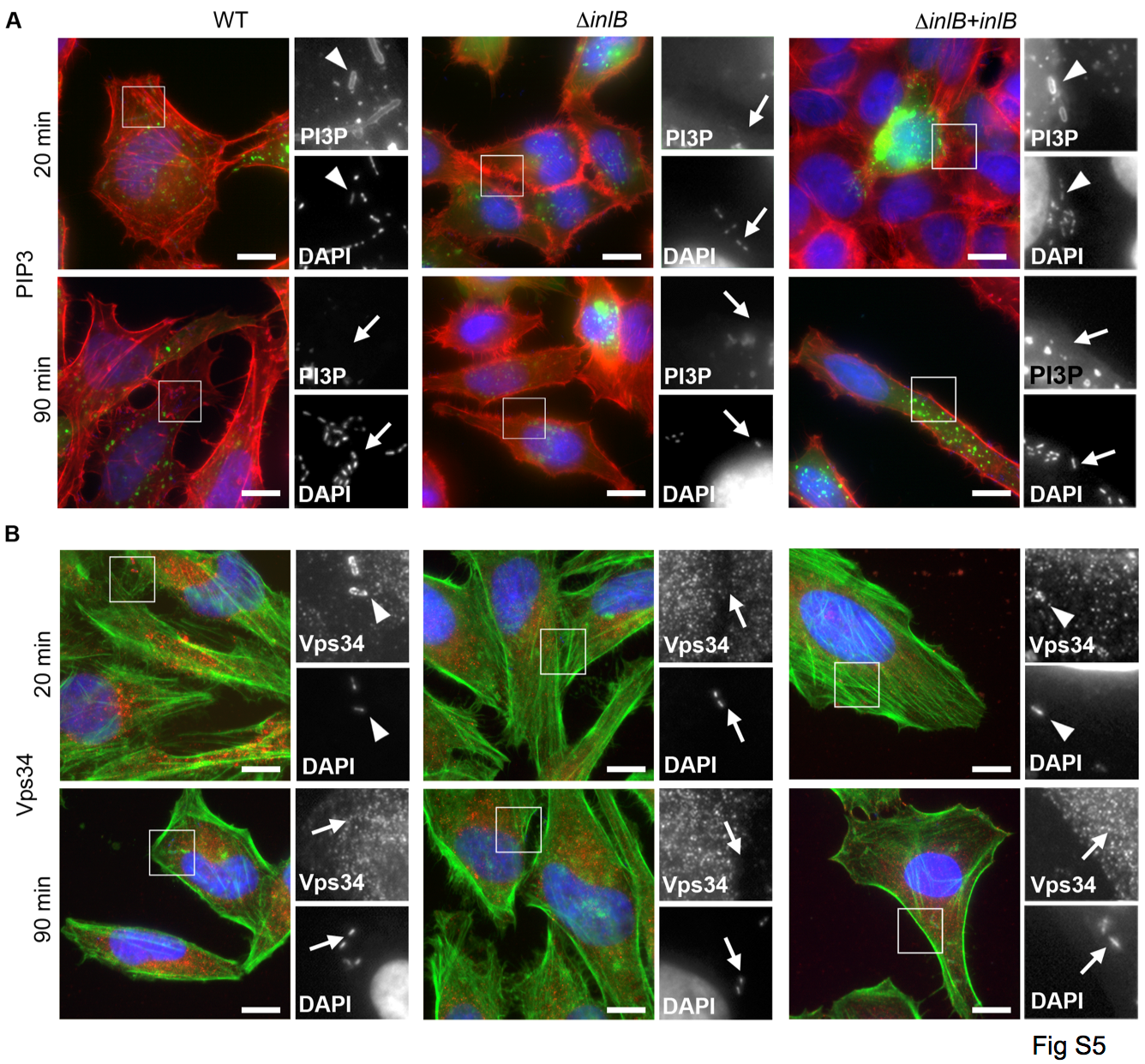

Supplement: FIG S5 [file mbio.03221-22-s0005.tif]

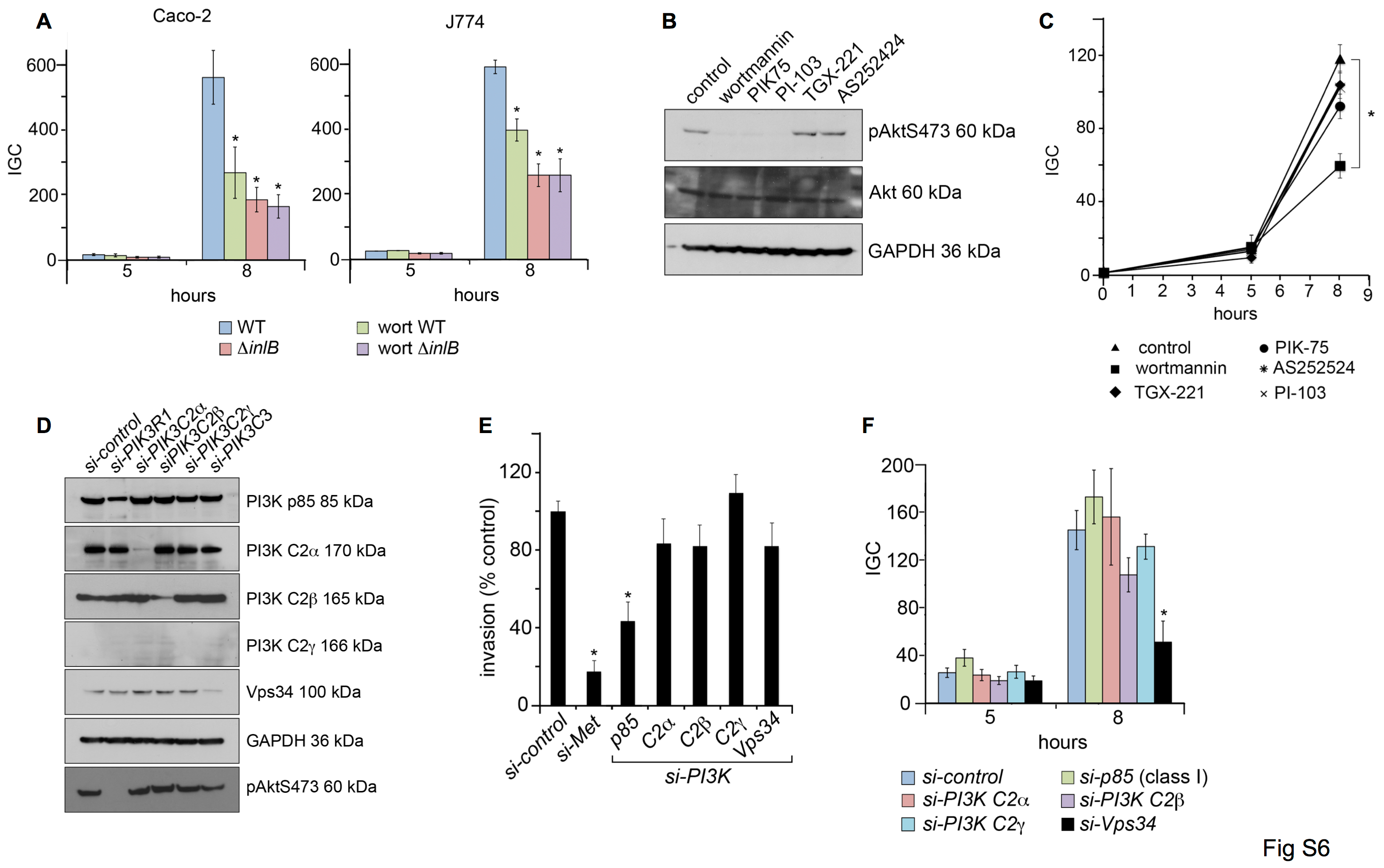

Supplement: FIG S6 [file mbio.03221-22-s0006.tif]

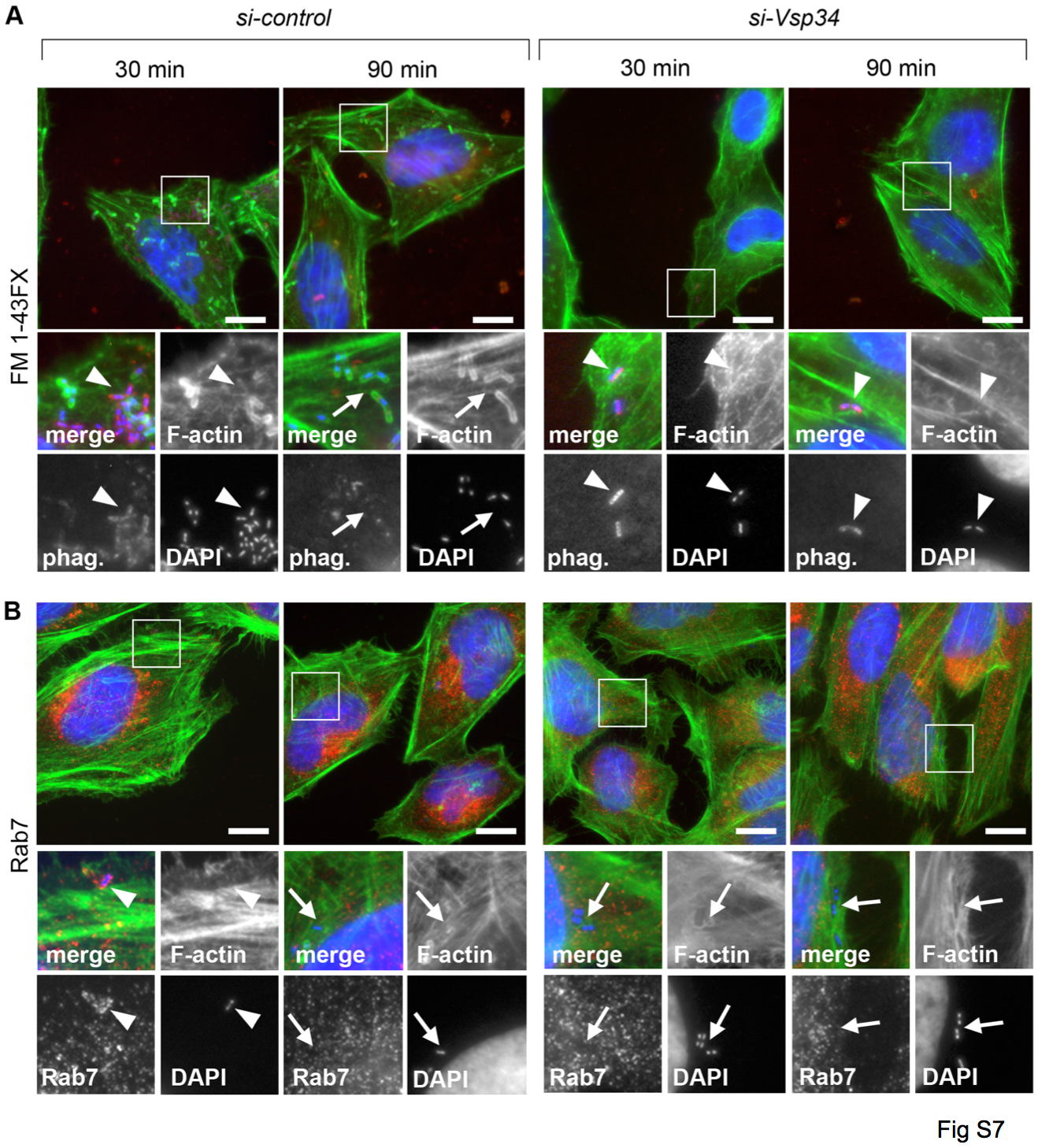

Supplement: FIG S7 [file mbio.03221-22-s0007.tif]

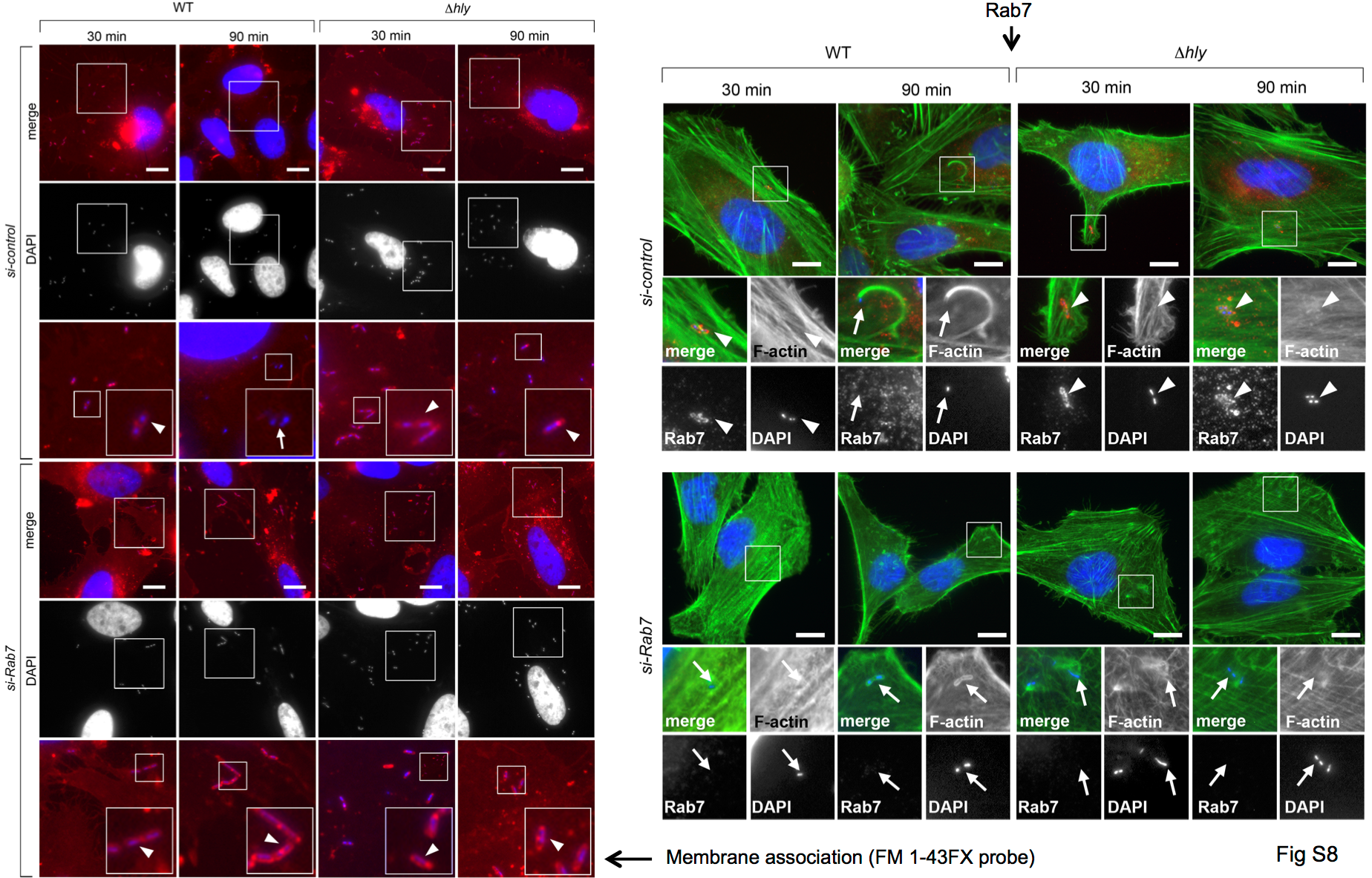

Supplement: FIG S8 [file mbio.03221-22-s0008.tif]

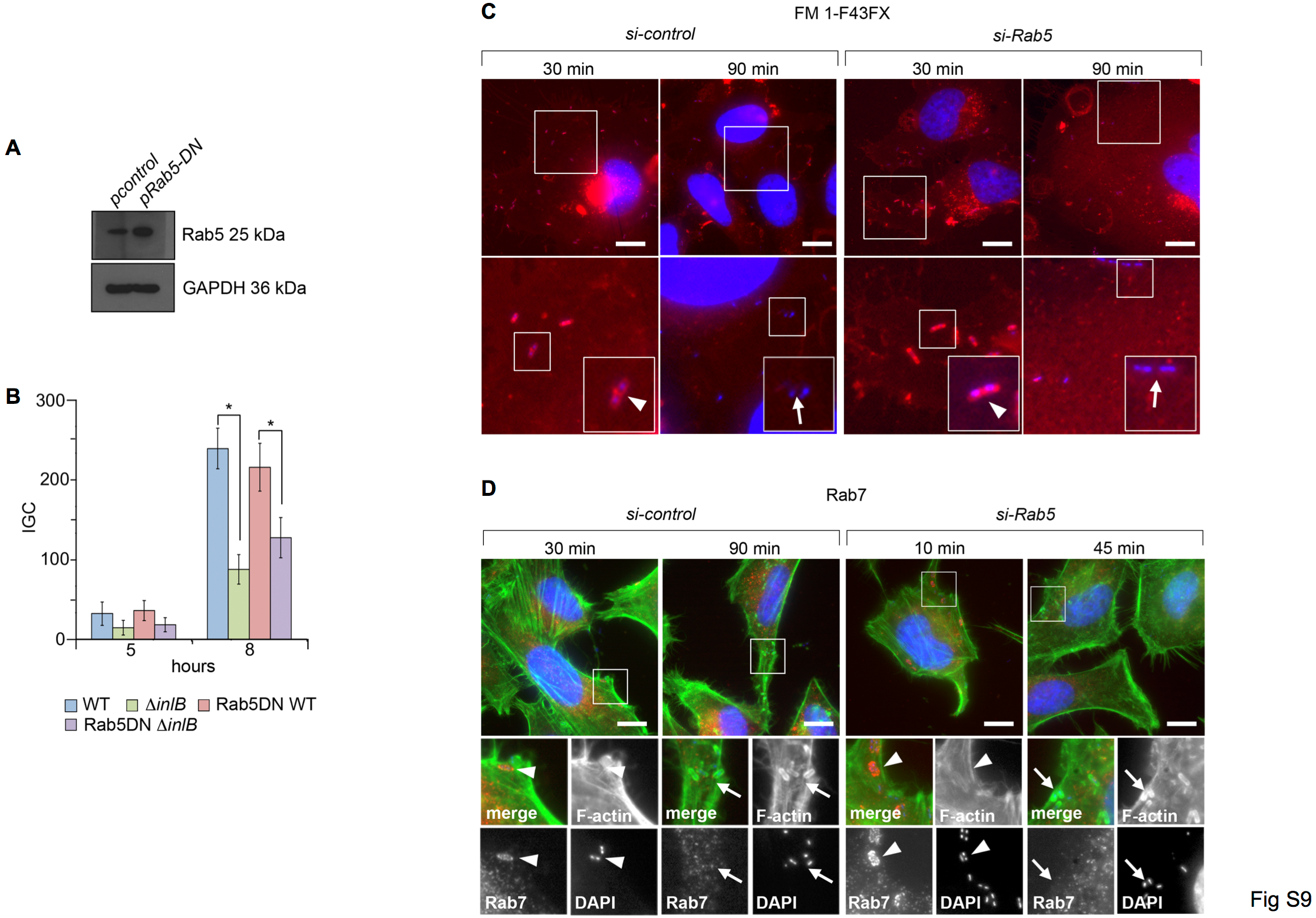

Supplement: FIG S9 [file mbio.03221-22-s0009.tif]

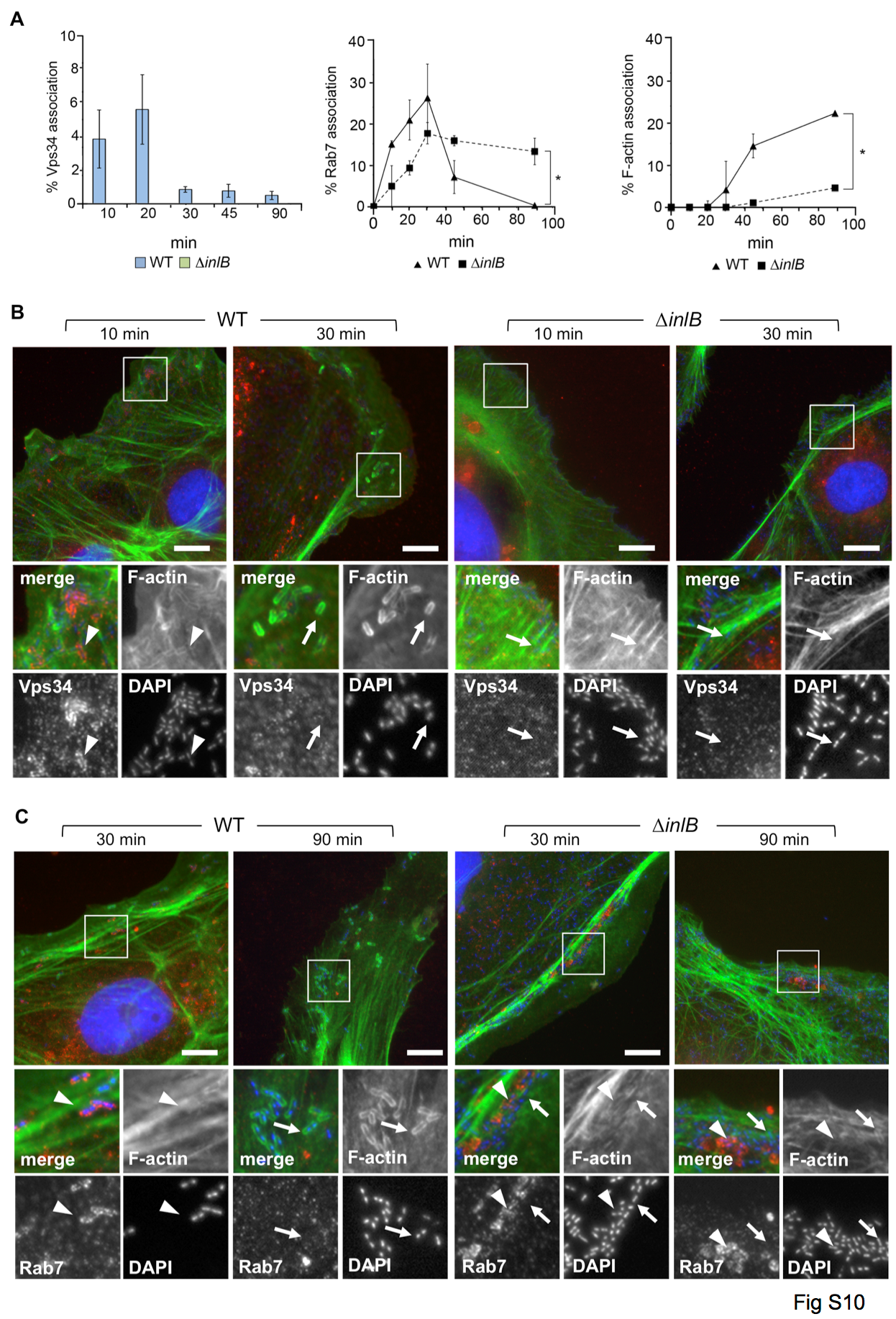

Supplement: FIG S10 [file mbio.03221-22-s0010.tif]
